# A State Monitoring Method for Shear Hinges in Floating Slab Tracks Based on FMTF and PCNN-RWKV Fusion Network

**DOI:** 10.3390/s26206446

**Published:** 2026-10-12

**Authors:** Zhenyu Wang, Jianming Zheng, Mingshun Yang

**Affiliations:** School of Mechanical Engineering, Xi’an University of Technology, Xi’an 710048, China

**Keywords:** floating slab track, shear hinge condition monitoring, Markov transition field, parallel convolutional neural network, Receptance Weighted Key Value

## Abstract

**Highlights:**

**What are the main findings?**

- A lightweight FMTF-PCNN-RWKV model is proposed for shear hinge monitoring, achieving 97.62% accuracy with strong robustness against noise and signal loss.
- FMTF extracts wideband features above 60 Hz; the model yields interpretable scattered attention, and fractured shear hinges show clear feature separation while normal/abnormal states have blurred boundaries.

**What are the implications of the main findings?**

- The lightweight model supports reliable shear hinge health monitoring under noisy engineering conditions.
- The identified feature rules provide insights for the intelligent structural fault diagnosis of shear hinges.

**Abstract:**

Floating slab track (FST) is a core vibration and noise reduction technology in urban rail transit. Shear hinges, key connectors of floating slabs, greatly affect rail transit operational stability and safety. Limited by complex operating conditions and weak feature extraction of traditional signal processing, existing methods yield inadequate feature representation, low diagnosis accuracy, and poor anti-noise performance. This study develops a novel shear hinge state recognition network combining Frequency Domain Markov Transition Field (FMTF), Parallel Convolutional Neural Network (PCNN), and Receptance Weighted Key Value (RWKV). Based on shear hinge working principles, vibration differences at floating slab ends indirectly reflect hinge health conditions. One-dimensional vibration spectrum sequences are transformed into 2D FMTF images to capture spectral dynamic transition features. PCNN extracts multi-scale image features, while RWKV fuses cross-channel dependencies for precise state identification. The model achieves accuracies of 98.19%, 95.39%, and 99.37% for normal, abnormal-response, and fracture states, respectively, with an overall accuracy of 97.62%. Featuring a compact architecture, it extracts multi-scale features efficiently with faster inference, offering a noise-resistant approach for FST shear hinge monitoring.

## 1. Introduction

As a core vibration and noise reduction technology for urban rail transit, the FST can mitigate vibration pollution caused by train operation, ensure riding comfort, and protect the surrounding environment, and has been widely applied in core urban sections and environmentally sensitive lines [1,2]. As a key component connecting adjacent floating slabs, the shear hinge undertakes the important functions of transferring shear forces, coordinating inter-slab deformations, and maintaining track alignment [3,4]. Under the action of complex coupled loads such as train dynamic loads and track irregularities, shear hinges are prone to damage such as cracking and loosening. If not detected and treated in a timely manner, such damage may lead to track irregularity and even derailment accidents [5]. Therefore, achieving early and accurate condition monitoring of shear hinges is an urgent engineering demand.

Health monitoring of FST is a prominent research hotspot in the fields of rail transit engineering and intelligent fault diagnosis. Scholars at home and abroad have conducted systematic investigations around three core directions: component dynamic characteristics, vibration signal feature extraction, and fault identification of key components [6,7]. At the component dynamics level, Hu et al. [3] revealed the force transmission mechanism of top-mounted shear hinges using the finite element submodeling technique. Wu et al. [5] and Fu et al. [8] verified that shear hinges can effectively constrain slab-end displacements and reduce vertical wheel–rail dynamic interactions. In the field of signal processing and fault diagnosis, Li et al. [9] completed the operational safety assessment of train–track–bridge coupled systems by combining Bayesian optimization and deep learning. Wang et al. [10] proposed a phase-coupled time–frequency analysis method to achieve high-precision identification of vibration isolator faults. However, most existing studies focus on the analysis of the forward dynamic mechanism of shear hinges, leaving obvious research gaps in the research on their inverse condition monitoring. Neither a quantitative mapping relationship between the vibration responses of floating slabs and the damage states of shear hinges has been established, nor have intelligent identification methods adapted to their cross-slab coupled vibration characteristics been developed.

The service state of shear hinges can be mapped to the coupled vibration responses of floating slabs at both ends through the inter-slab shear force transmission path, and this indirect measurement method faces three fundamental challenges [11,12,13]. First, the vibration responses contain coupled vertical, lateral, and longitudinal components. Thus, unidirectional measurement cannot fully characterize the inter-slab shear transfer characteristics. Second, the damage feature energy is diffusely distributed over a wide frequency band, and significant nonlinear abrupt variations occur before and after shear hinge fracture, rendering traditional linear measurement models ineffective. Third, the 25-year design service life of shear hinges leads to extremely weak damage signals, while complex operating conditions induce strong non-stationary vibration fluctuations, which severely degrade the signal-to-noise ratio and measurement repeatability.

Feature extraction and model construction constitute the core of shear hinge service state measurement based on vibration signals [14]. In terms of feature extraction, scholars have integrated time–frequency analysis algorithms and deep learning models to conduct fault diagnosis research [15,16,17]. Methods including Gramian Angular Field (GAF) [18], Markov Transition Field (MTF) [19], Continuous Wavelet Transform (CWT) [20], and Short-Time Fourier Transform (STFT) [21] can convert one-dimensional time series into two-dimensional matrices applicable to CNN input. Nevertheless, these methods have inherent limitations, such as only capturing linear relationships, failing to characterize frequency component coupling, and introducing temporal redundancy. Time–frequency fusion methods partially compensate for these deficiencies [22], but most are designed for rotating machinery and thus inapplicable to non-rotating components such as shear hinges, whose damage features are widely dispersed across broad frequency bands [23].

In terms of model construction, extracting damage-sensitive features from high-dimensional non-stationary responses and establishing reliable mapping relationships is a core scientific problem in structural measurement. Hybrid architectures that combine the local spatial feature extraction capability of CNNs with the global temporal modeling capability of RNNs or Transformers have become mainstream solutions [24,25]. To enhance the multi-scale extraction capability for wideband diffuse damage features, researchers have introduced the Efficient Layer Aggregation Network (ELAN) and its variants in computer vision into structural health monitoring tasks [26,27,28,29]. However, most ELAN-based methods are designed for single-channel signal analysis [30,31] and cannot capture cross-channel coupling relationships, which are critical for shear hinge state measurement. To fully extract fusion features from multi-component signals, emerging temporal models such as RWKV [32,33,34,35] exhibit superior performance in balancing long-sequence dependency capture and computational efficiency. These models avoid the gradient vanishing problem and high computational complexity of traditional methods, enabling potential edge deployment. Existing studies have applied RWKV to scenarios including cross-modal feature fusion [36,37] and multi-semantic interaction [38], verifying its advantages in feature fusion. Nevertheless, most of these studies focus on general data fusion without physical constraints and lack targeted optimization for multi-channel vibration signals with strong physical coupling characteristics.

To address the above limitations, this paper takes vibration signals collected from two floating slabs as the data source and proposes a shear hinge condition monitoring method based on an FMTF-driven PCNN-RWKV fusion network. The core innovations and contributions of this study are summarized as follows:

(1) A frequency-domain correlation enhancement method termed FMTF is proposed to adapt to the spectral characteristics of shear hinges. The proposed method converts vibration spectra into two-dimensional matrices and constructs global frequency-domain correlations based on transition probability characteristics.
(2) A PCNN-RWKV fusion neural network tailored to the multi-component FMTF features of shear hinges is developed. PCNN is employed to extract multi-scale features from multi-component FMTF images, and the obtained multi-scale features are input into the RWKV model to capture cross-channel coupling features and complete shear hinge state recognition.
(3) The proposed FMTF-based PCNN-RWKV shear hinge state recognition model achieves high recognition accuracy with fewer parameters and lower floating-point operations on multiple metro lines, realizing precise and reliable state identification of shear hinges.

The remainder of this paper is organized as follows: Section 2 elaborates on the working principle of shear hinges and the configuration of the condition monitoring system; Section 3 details the architecture and core module design of the FMTF and PCNN-RWKV-based fusion network; Section 4 verifies the comprehensive performance of the proposed model via comparative experiments; Section 5 summarizes the research findings and outlines future research directions.

## 2. Working Principle and Condition Monitoring System Construction

In this section, to accurately identify the working state of shear hinges in FST, a condition monitoring system for shear hinges is established through the analysis of their actual working principle and fault states.

### 2.1. Working Principle and Fault Analysis of Shear Hinges

**Figure 1:**
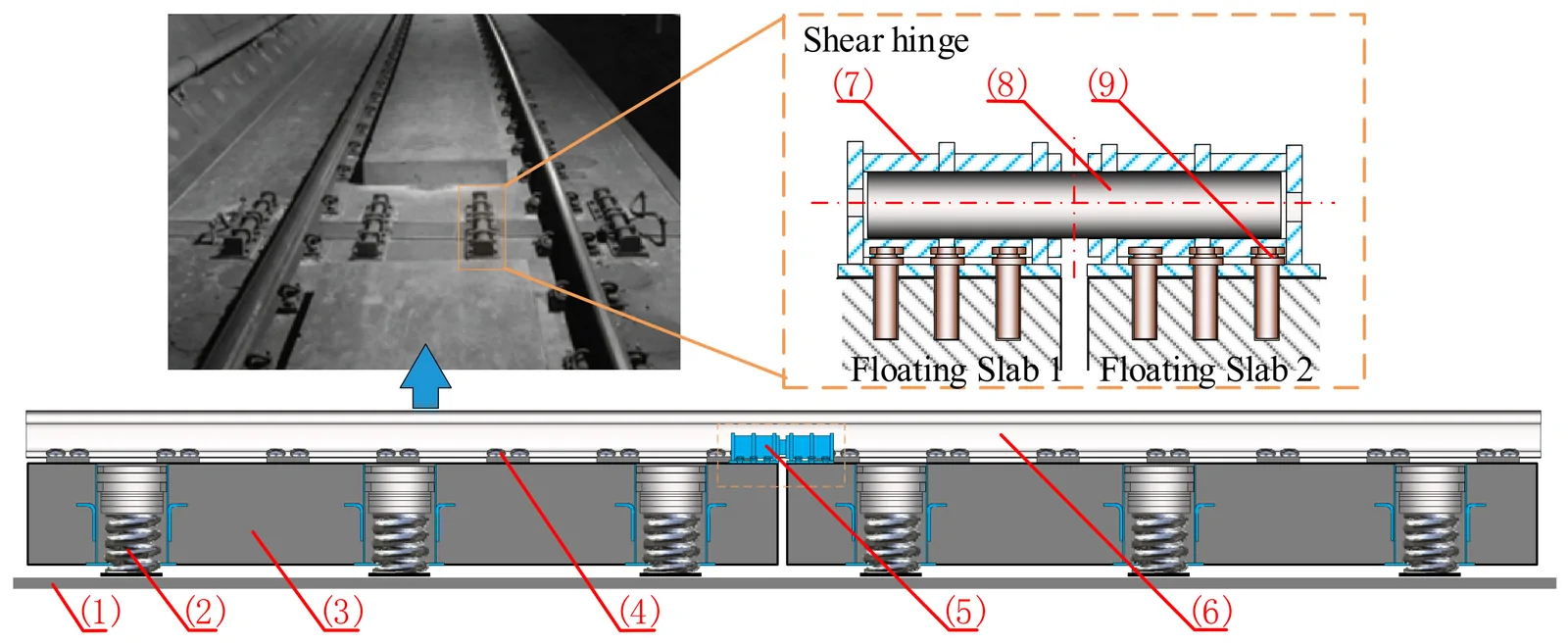
Working principle and structural schematic diagram of FST: (1) Foundation, (2) Steel spring isolator, (3) Floating slab, (4) Fastener, (5) Shear hinge, (6) Rail, (7) Hinge seat, (8) Shear dowel, (9) Bolts.

An FST is composed of steel rails, fasteners, floating slabs, steel spring isolators, shear hinges, and the foundation, as shown in Figure 1.

When a metro train passes by, the dynamic load generated by the train is transmitted to the floating slabs through the rail–fastener system, and then from the floating slabs to the concrete foundation via the steel spring isolator array. Floating slabs are connected by shear hinges, allowing rail loads to transfer between floating slabs through the shear hinges.

**Figure 2:**
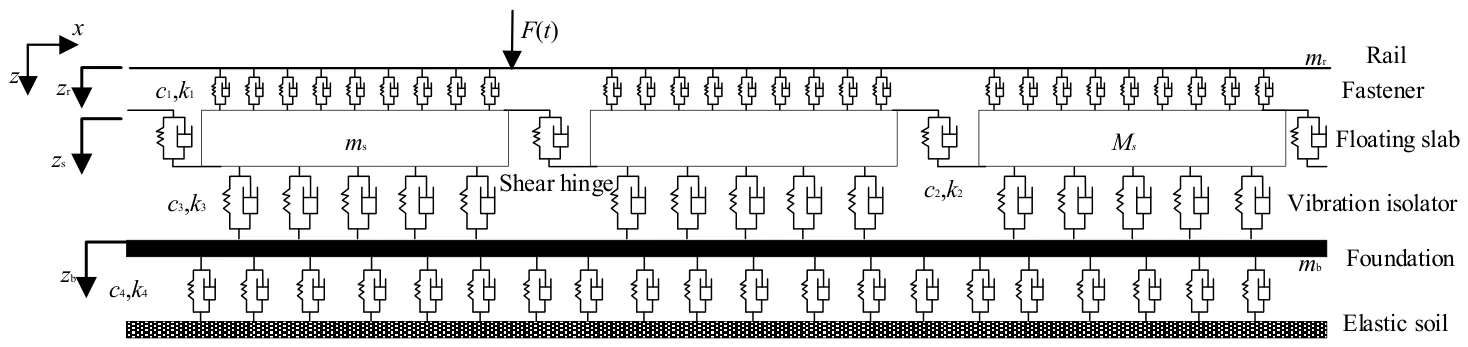
FST Mathematical Model.

Based on vehicle–track coupled dynamics, the influence of the shear hinge system on the dynamic characteristics of slab tracks is analyzed below. The simplified model of the FST with shear hinges established in Reference [5] is presented in Figure 2.

The magnitude of the shear force exerted by shear hinges is primarily determined by the displacement difference between the two adjacent floating slabs; the larger the displacement difference, the greater the shear force acting on the shear hinges. When the stiffness and damping beneath the two floating slabs are consistent, the displacement difference between them caused by passing trains is negligible, and the generated vibrations are similar; in this case, the shear hinges are in a normal service state. When there are differences in the conditions of the two floating slabs, the displacement difference caused by passing trains increases, and the generated vibrations become inconsistent, leading to an increase in the shear force on the hinges; at this point, the shear hinges are in an abnormal-response state. Prolonged abnormal response will accelerate the fracture of shear hinges. When a shear hinge fractures, the continuity of the floating slabs is destroyed, and energy propagates directly forward along the rails. The displacement difference between the two floating slabs still exists when trains pass through, and the vibrations of the two slabs are significantly greater than those under normal conditions.

### 2.2. Construction of Shear Hinge Condition Monitoring System

**Figure 3:**
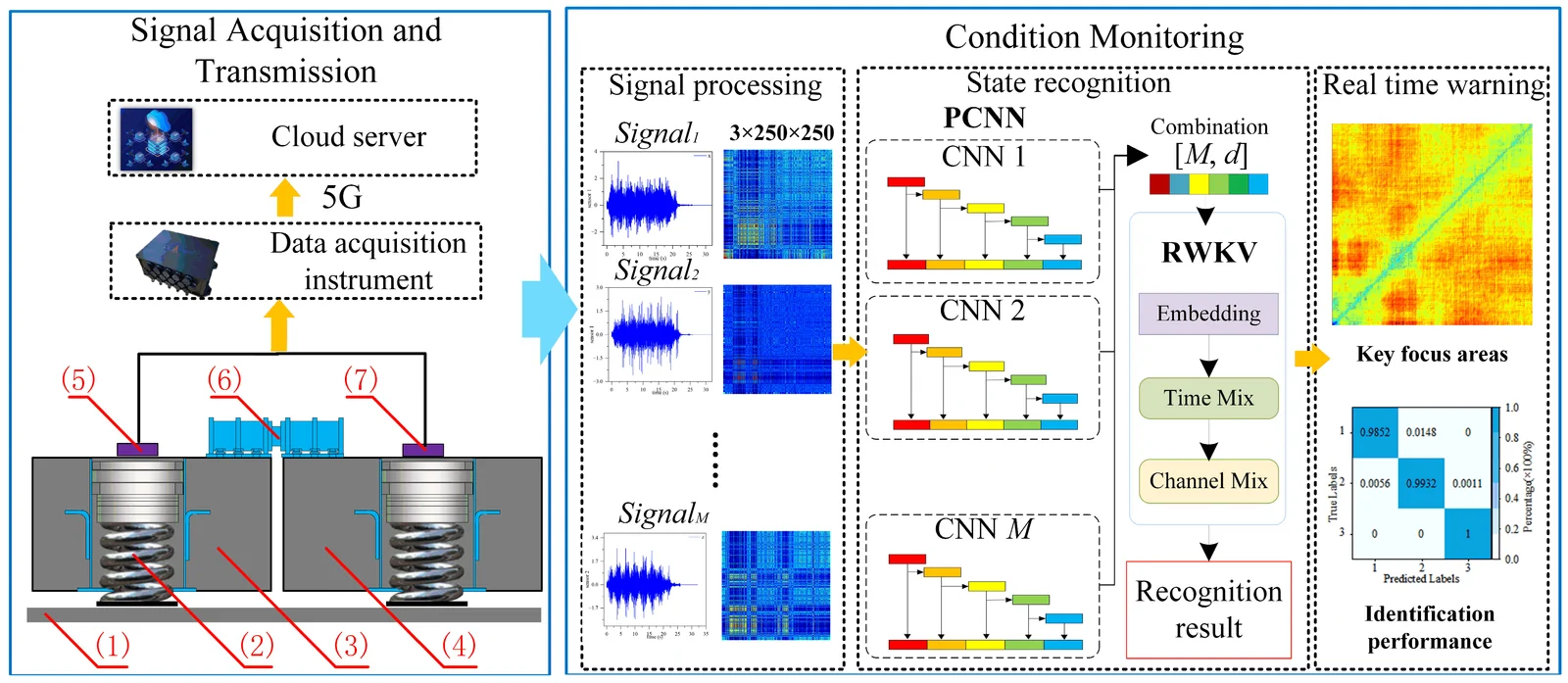
Sensor layout scheme for online monitoring system of FST shear hinge: (1) Foundation, (2) Vibration isolator, (3) Floating slab 1, (4) Floating slab 2, (5) Triaxial accelerometer 1, (6) Shear dowel, (7) Triaxial accelerometer 2.

Constrained by the mechanical characteristics and installation conditions of shear hinges, direct measurement of shear force signals is difficult. To address this issue, vibration signals of floating slabs at both ends of the shear hinge are acquired via vibration sensors in engineering practice to indirectly monitor the shear hinge state. In accordance with the operating principle of shear hinges, the established online monitoring system employs two triaxial accelerometers to simultaneously capture three-directional vibration signals of the floating slabs at both ends of the shear hinge, as shown in Figure 3.

## 3. Proposed FMTF-PCNN-RWKV

**Figure 4:**
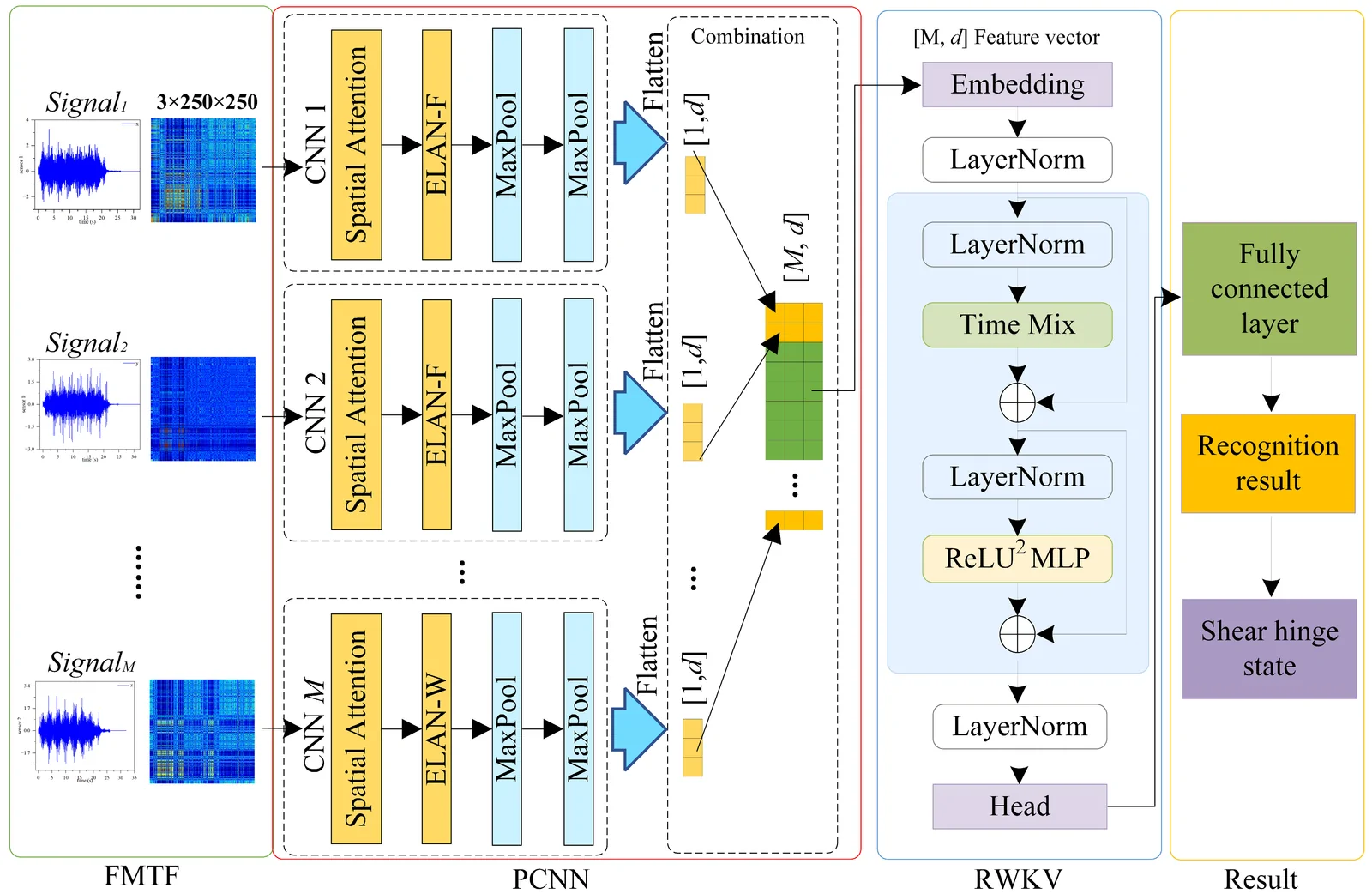
PCNN-RWKV Convergence Network Framework Based on FMTF.

Due to the complex service conditions of shear hinges, the difficulty in directly measuring their physical characteristics, and the inability of traditional signal processing methods to effectively extract their state features, state recognition models suffer from challenges such as difficulty in effective feature extraction, low accuracy, and poor generalization ability. To this end, this paper proposes a deep learning model based on the FMTF for shear hinge state recognition. The overall architecture of the model is shown in Figure 4, which mainly consists of three modules: frequency-domain global correlation enhancement based on the MTF, multi-scale feature extraction based on a PCNN, and cross-channel dependency modeling based on receptance weighted key-value mechanisms.

(1) Frequency-domain global correlation enhancement based on MTF. When a train passes the shear hinge, vibration signals of the floating slabs at both ends of the shear hinge are obtained. A Markov chain is used to extract the MTF from the vibration signal spectra of the floating slabs, enhancing global frequency-domain correlation and long-range dependencies to provide feature support for shear hinge state recognition.
(2) Multi-scale feature extraction based on PCNN. To address the characteristics of shear hinge FMTF, a PCNN adapted to multi-component FMTF of shear hinges is proposed, which integrates a spatial attention mechanism and an efficient aggregation network to simultaneously extract multi-scale features from multi-component FMTF, improving the feature extraction capability of the model.
(3) This section establishes cross-channel dependencies based on RWKV. Taking the multi-scale features extracted by PCNN as input, it integrates the RWKV mechanism to address the inherent limitations of PCNN in capturing global frequency features and cross-sensor features, further enhancing the model’s cross-channel feature fusion capability. Finally, the features extracted by RWKV are fed into a fully connected network to output the final recognition results.

### 3.1. FMTF

The FMTF converts 1D vibration spectrum sequences into 2D FMTF based on the state transition probability of the Markov process, enabling the characterization of dynamic patterns of state transitions within spectrum sequences. The core of the FMTF lies in establishing state transition relationships between different frequencies based on Markov theory.

First, the spectrum sequence *S* = {*X*_0_, *X*_1_, …, *X*_m_} is sorted in descending order to obtain the sequence *S*_sort_. Then, *S*_sort_ is divided equally into *Q* states of equal length. For each state *q*, there are *Q* possible transitions to other states. However, in an actual spectrum, state *q* transitions to only *u* states, *u* ≤ *Q*, meaning that the probability of transitioning to these *u* states is greater.

Each frequency point of the spectrum sequence *S* has a corresponding state, meaning that the original spectrum sequence can be transformed into a 1 × *m* state sequence $S_{Q}=\{q_{X_{0}},q_{X_{1}},\cdots ,q_{\mathrm{Xm}}\}$. By counting the number of transitions from each state *q* to other states in *S_Q_*, a *Q* × *Q* Markov transition matrix **W** can be constructed. In the original spectrum sequence, there is no correlation between different frequencies. To enhance this global frequency correlation, based on the Markov transition matrix **W**, the transition probability from each frequency point to *m* frequencies is calculated, thereby forming an *m* × *m* FMTF **M** as shown in Equation (1).

(1) $$M=\left(\begin{array}{ccc} m_{\mathrm{ij}|(X_{1}\in q_{i},X_{1}\in q_{j})} & \cdots & m_{\mathrm{ij}|(X_{1}\in q_{i},X_{n}\in q_{j})}\\ m_{\mathrm{ij}|(X_{2}\in q_{i},X_{1}\in q_{j})} & \cdots & m_{\mathrm{ij}|(X_{2}\in q_{i},X_{n}\in q_{j})}\\ \vdots & & \vdots \\ m_{\mathrm{ij}|(X_{n}\in q_{i},X_{1}\in q_{j})} & \cdots & m_{\mathrm{ij}|(X_{n}\in q_{i},X_{n}\in q_{j})} \end{array}\right),$$

where mij denotes the transition probability that quantile qi follows quantile qj, i.e., *m_ij_* = *P*(*q_i_* → *q_j_*), where *i*,*j* ∈ [1,*Q*].

### 3.2. PCNN

Considering the characteristics of shear hinge vibration signals, a PCNN adapted for multi-channel and cross-sensor scenarios is designed to fully extract the multi-scale features of the multi-component FMTF.

**Figure 5:**
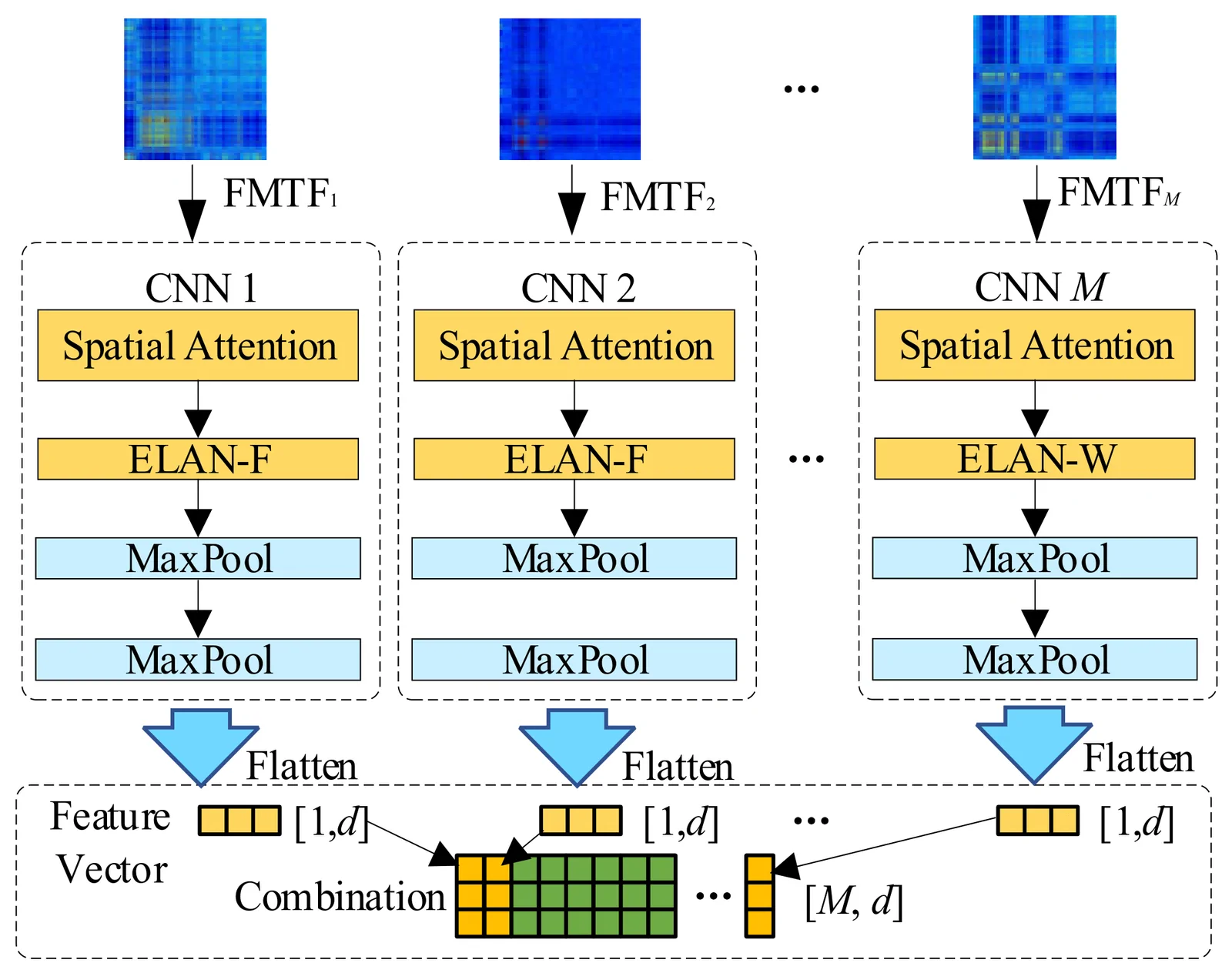
Structure of PCNN.

PCNN accounts for the multi-component FMTF characteristics of shear hinge states and improves the model’s feature extraction capability at the structural level through targeted architecture and module design. The structure of PCNN is shown in Figure 5, and its structural features include a parallel multi-branch architecture, spatial attention mechanism, and multi-scale feature extraction.

#### 3.2.1. Parallel Multi-Branch Architecture

The parallel multi-branch architecture refers to designing the feature extraction of multi-component FMTF as parallel paths. When M image features of the same size need to be extracted, the inputs of the M parallel CNNs are as shown in Equation (2).

(2) $$\chi ={\left\{I^{g}\right\}}_{g=1}^{G},$$

where M is the total number of parallel CNNs. represents the input image tensor of the g-th parallel CNN, where C denotes the number of channels, H is the height of the feature map, and W is the width of the feature map. This parallel design can effectively extract multi-scale features of a single-component FMTF while avoiding feature loss.

#### 3.2.2. Spatial Attention

Spatial Attention refers to focusing on the regions in the FMTF that are strongly correlated with the shear hinge state, while weakening the noise interference from irrelevant regions. Before the global convolution of the 2DCNN, it makes the features more focused on the key frequency domains for state recognition, directly increasing the proportion of effective information in the features and facilitating subsequent feature extraction.

The mean value $I_{\mathrm{avg}}$ and maximum value $I_{\max}$ along the channel dimension are calculated separately for the input feature map $I^{g}$, as shown in Equations (3) and (4).

(3) $$I_{\mathrm{avg}}=E_{c}(I_{t}^{g})=\frac{1}{C}\sum_{c=1}^{C}I_{t,c}^{g}\in R^{1\times H\times W},$$

(4) $$I_{\max}=\max_{c}(I_{t}^{m})=\max_{1\leq c\leq C}I_{t,c,:,:}^{m}\in R^{1\times H\times W},$$

The mean and maximum features are concatenated along the channel dimension, as shown in Equation (5).

*I*_concat_ = concat(*I_avg_*,*I_max_*) ∈ *ℝ*^2×*H*×*W*^, (5)

The spatial attention weights are learned through convolution, and the spatial attention matrix is obtained as shown in Equation (6).

*A*_raw_ = conv_2→1_(*I*_concat_) ∈ *ℝ*^1×*H*×*W*^, (6)

The weights are mapped to the range [0,1] using the Sigmoid function, yielding the attention map A as shown in Equation (7).

*A* = *σ*(*A*_raw_) ∈ *ℝ^B^*^×1×*H*×*W*^, (7)

The attention map is multiplied element-wise with the original feature to obtain the weighted feature map $I_{t,\mathrm{SA}}^{g}$, as shown in Equation (8).

(8) $$I_{t,\mathrm{SA}}^{g}=I_{t}^{g}⊙A,$$

**Figure 6:**
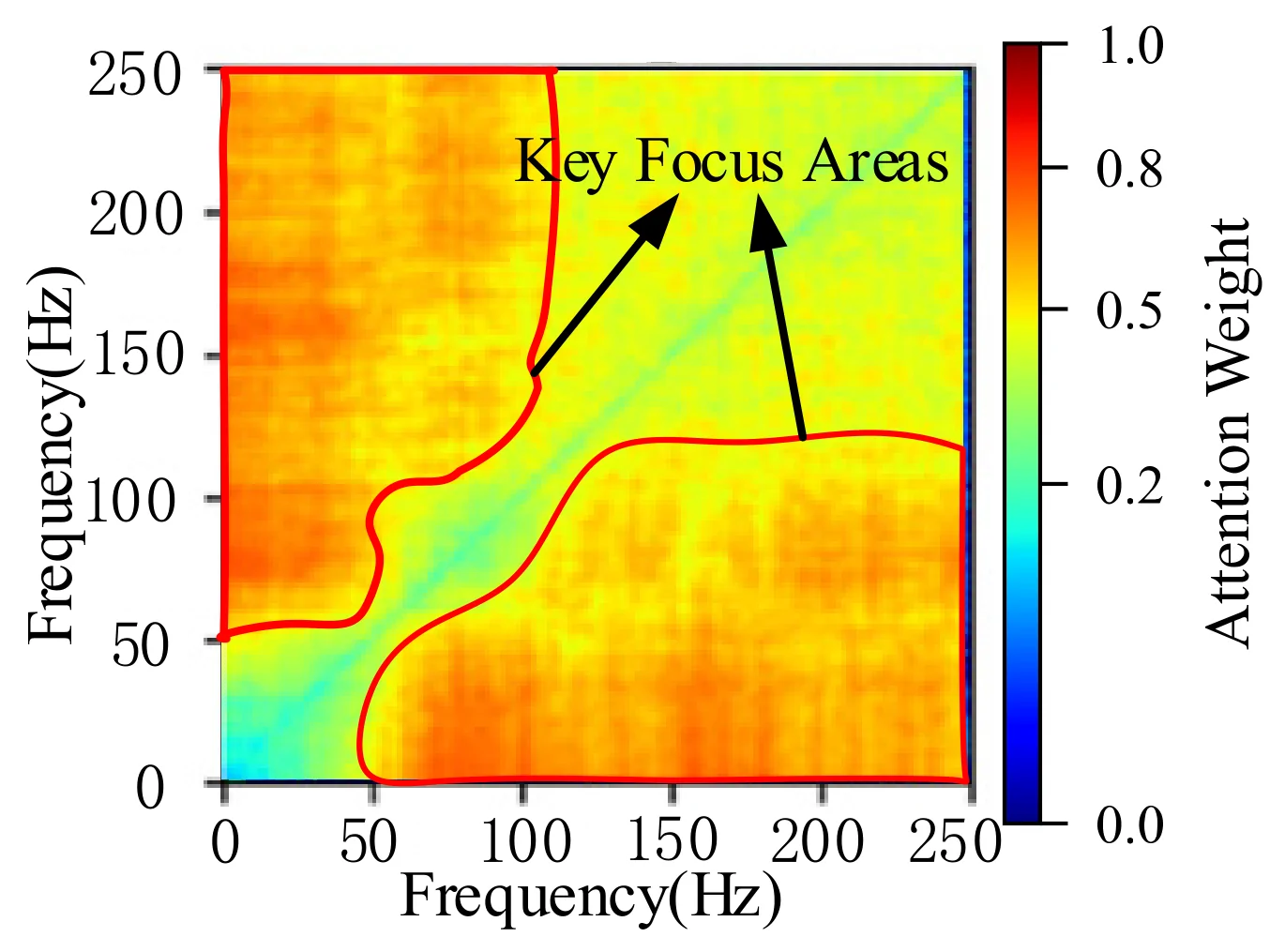
Focus areas of X-axis of multiple actual metro lines.

When the state of the shear hinge changes, corresponding regional variations can be observed in the FMTF. The red-boxed areas in Figure 6 represent the key regions of interest for X-axis data from multiple actual metro lines. The frequency band of 60–250 Hz is where the critical features associated with shear hinge state variations are concentrated.

#### 3.2.3. Multi-Scale Feature Extraction

**Figure 7:**
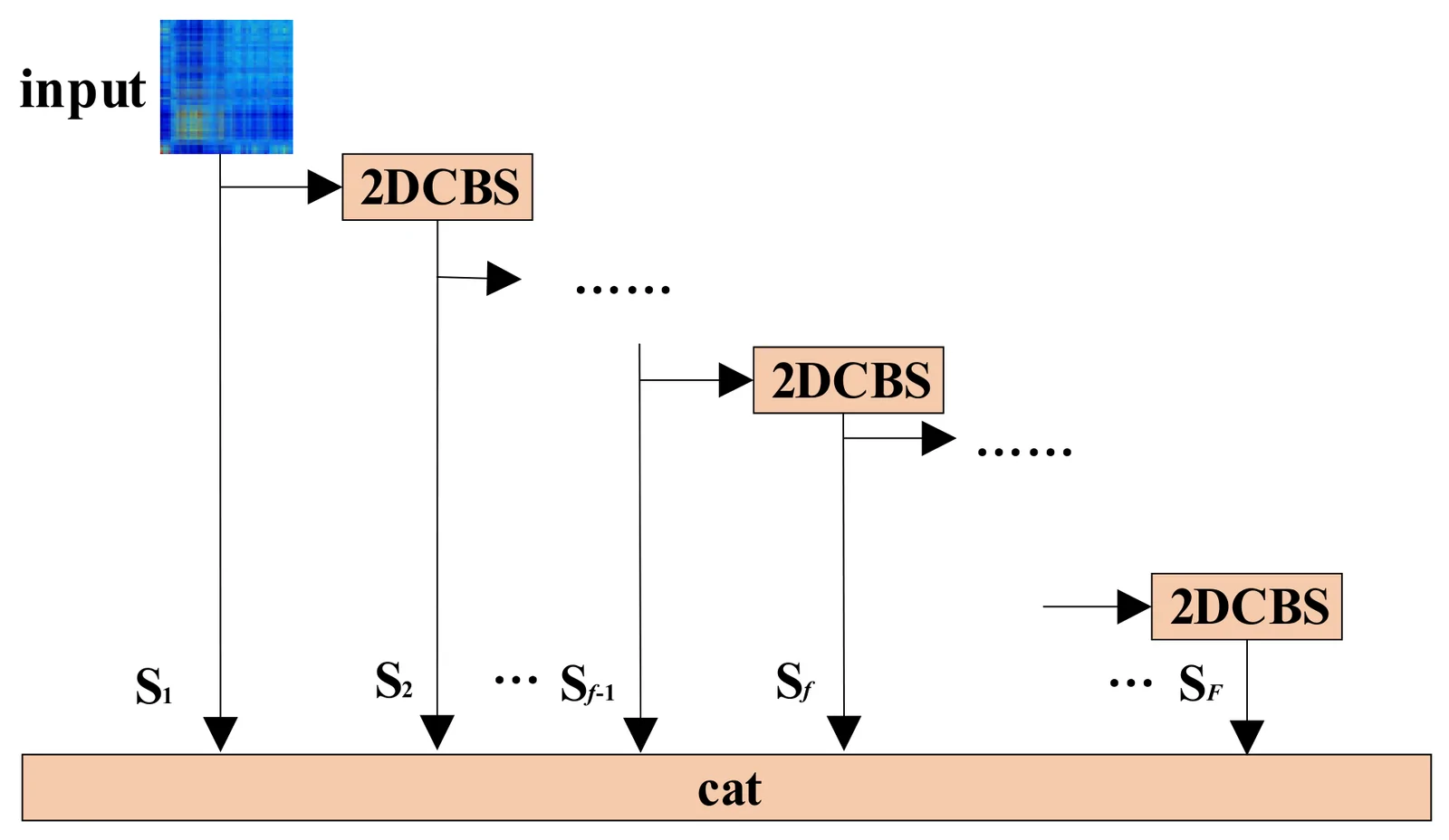
ELAN-F network structure.

Multi-scale feature extraction refers to drawing on the efficient aggregation network (ELAN) and adopting feature enhancement strategies such as dilation, shuffling, and merging to improve the ability of 2DCNN to extract multi-level features, including low-dimensional local textures, medium-dimensional structural patterns, and high-dimensional abstract states [27]. Compared with the traditional convolutional neural network with a single-branch structure, the multi-scale features of the ELAN-F module are generated by concatenating multi-branch features. It can fully characterize the complex features of the FMTF, significantly improving the feature representation ability, and finally producing the output through feature combination, as shown in Figure 7.

In the multi-scale feature generation and concatenation process of ELAN-F, the input feature map of the ELAN-F layer is denoted as $I_{t,\mathrm{SA}}^{g}$. ELAN-F generates F feature maps of different scales [S_1_, S_2_, …, S*_f_*_−1_, S*_f_*, …, S*_F_*] via F parallel 2DCBS branches, where F is the core parameter of the ELAN-F module. The feature of the f-th branch is given in Equation (9).

(9) $$S_{f}^{t}=2{\mathrm{DCBS}}^{f}(I_{t,\mathrm{SA}}^{m}),$$

Here, 2DCBS*^f^* denotes that the *f*-th branch undergoes an *f*-level 2DCBS transformation to obtain the corresponding scale feature. 2DCBS consists of a two-dimensional convolution, a batch normalization layer (BN), and the SiLU activation function. Its transformation on the input feature map $I_{t,\mathrm{SA}}^{g}\in R^{C\times H\times W}$ is shown in Equation (10).

(10) $$2\mathrm{DCBS}(I_{t,\mathrm{SA}}^{m})=\mathrm{SiLU}\left(\mathrm{BN}\left({\mathrm{conv}}_{3\times 3}(I_{t,\mathrm{SA}}^{m})\right)\right),$$

where Conv_3×3_ represents a 2D convolution with a kernel size of 3 × 3 and a stride of 1. BN denotes the batch normalization layer, which standardizes the convolution output. SiLU(x) = x*σ*(x), where *σ* represents the Sigmoid function.

Each branch has a different number of output channels. Let the number of input channels be C_in_, and the number of output channels of the *f*-th branch be C*_f_*, as shown in Equation (11).

(11) $$C_{f}=\max\left(\left\lfloor \frac{C_{\mathrm{in}}}{2^{f-1}}\right\rfloor ,\;1\right),\;\;\;\;f=1,2,\ldots ,F,$$

Subsequently, all multi-scale features are concatenated along the channel dimension to obtain the output $I_{(t)\mathrm{SF}}^{F}$ of this layer, as shown in Equation (12).

(12) $$I_{t,\mathrm{SF}}^{g}=\mathrm{cat}(S_{1}^{t},S_{2}^{t},\cdots ,S_{F}^{t}),$$

If the output of each branch is $S_{f}^{t}\in R^{C_{f}\times H\times W}$, the dimension after concatenation is $S_{F}^{t}\in R^{C_{F}\times H\times W}$, which serves as the input to the next layer, with $C_{F}=\sum_{f=1}^{F}C_{f}$.

After feature extraction by ELAN-F, the dimension of the feature vector $I_{t,\mathrm{SF}}^{F}$ increases. It is gradually down-sampled through *b* max-pooling layers to obtain the down-sampled feature vector $I_{t,\mathrm{MP}}^{F}$, as shown in Equation (13).

(13) $$I_{t,\mathrm{MP}}^{m}={\mathrm{MaxPool}}_{2\times 2}^{b}(I_{t,\mathrm{SF}}^{F}),$$

where MaxPool denotes max-pooling. The feature map $I_{t,\mathrm{MP}}^{F}$ is flattened into a two-dimensional feature vector, which is then regularized using Dropout to obtain the output of the current CNN, as shown in Equation (14).

(14) $$I_{t,\mathrm{PCNN}}^{g}=\mathrm{dropout}(\mathrm{flatten}(I_{t,\mathrm{MP}}^{F})\in R^{1\times D},p),$$

All parallel outputs $I_{t,\mathrm{PCNN}}^{g}$ are merged as different channels into a feature vector $I_{t,\mathrm{PCNN}}^{G}\in R^{M\times D}$, thereby obtaining multi-scale key features that represent the multi-component FMTF, providing a multi-scale feature basis for subsequent state recognition.

### 3.3. RWKV

Based on the multi-level features extracted by PCNN, this section integrates the RWKV to overcome the limitations of PCNN in extracting global frequency-domain features and cross-sensor features. PCNN can effectively extract multi-scale frequency-domain features from a single-component FMTF. However, extracting features from multi-component fields requires larger convolution kernels, such as 3D convolution kernels, which leads to a sharp increase in computational cost and is unfavorable for improving the overall performance of the model. Integrating the multi-scale feature extraction capability of PCNN with the cross-channel feature extraction capability of RWKV further enhances the comprehensive recognition performance of the proposed model.

The time-mixing and channel-mixing modules of the RWKV7 [39] model serve as its core components for global and multi-channel feature extraction. They combine the advantages of Transformer’s temporal modeling capability and RNN’s computational efficiency, achieving outstanding performance in various sequence tasks.

Compared with the attention mechanism in Transformer, the time-mixing module of RWKV7 adopts the generalized incremental rule combined with a flexible vector gating mechanism. This not only reduces computational complexity but also delivers powerful dynamic-state recognition capability, and its calculation formula is shown in Equation (15).

(15) $$S_{t}=S_{t-1}\left(\mathrm{diag}(w_{t})-{\hat{\kappa }}_{t}^{⊤}(a_{t}⊙{\hat{\kappa }}_{t})\right)+v_{t}^{⊤}{\tilde{k}}_{t},$$

where *w_t_* ∈ *ℝ^D^* is the vector-valued decay factor, *a_t_* ∈ (0,1)*^D^* denotes the in-sequence learning rate, and ${\hat{\kappa }}_{t},{\tilde{k}}_{t}\in R^{D}$ represents the normalized remove key and replace key, respectively. This design decouples the key ${\hat{\kappa }}_{t}=\kappa_{t}/\|\kappa_{t}{\|}_{2}$ for state removal and the key ${\tilde{k}}_{t}=k_{t}⊙\mathrm{lerp}(1,a_{t},\alpha )$ for replacement, enabling per-channel control of dynamic states.

The weight preparation module generates adaptive parameters through a low-rank MLP to balance efficiency and expressiveness, as shown in Equation (16).

(16) $$\begin{array}{cc} a_{t} & =\mathrm{sigmoid}\left({\mathrm{loramlp}}_{a}(x_{t}^{a})\right),\;\;\;\;(\mathrm{In-context}\;\mathrm{Learning}\;\mathrm{Rate})\\ w_{t} & =\exp\left(-e^{-0.5\mathrm{sigmoid}(d_{t})}\right),\;\;\;\;(\mathrm{Dynamic}\;\mathrm{Decay}\;\mathrm{Factor})\;\\ \kappa_{t} & =k_{t}⊙\xi ,\;{\tilde{k}}_{t}=k_{t}⊙(1-a_{t}⊙\alpha )\;\;\;\;(\mathrm{Key}\;\mathrm{Generation}) \end{array},$$

where *ξ* ∈ *ℝ^D^* and *α* ∈ *ℝ^D^* are learnable parameters, used for scaling the remove key and the replacement rate, respectively. Different from the scalar decay in RWKV6, the vector-valued *w*_t_ and *a*_t_ in the RWKV7 model support independent per-channel tuning, which enhances the model’s ability to model complex sequential patterns.

The channel mixing module of RWKV7 no longer adopts the one from RWKV6. By removing redundant gating, expanding the hidden dimension, and introducing time-series interpolation, it improves computational efficiency and parallelism while maintaining parameter efficiency and representation capability, preserving the core advantage of the RWKV series: efficient sequence modeling. The features extracted by RWKV are mapped to classification labels via a fully connected layer.

## 4. Case Study

### 4.1. Experimental Data

To verify the effectiveness of the model, multiple operating lines were monitored based on the above monitoring scheme. A large number of shear hinge state signals were collected through signal acquisition, and a shear hinge state dataset was constructed for validating the model performance.

#### 4.1.1. Signal Acquisition

**Figure 8:**
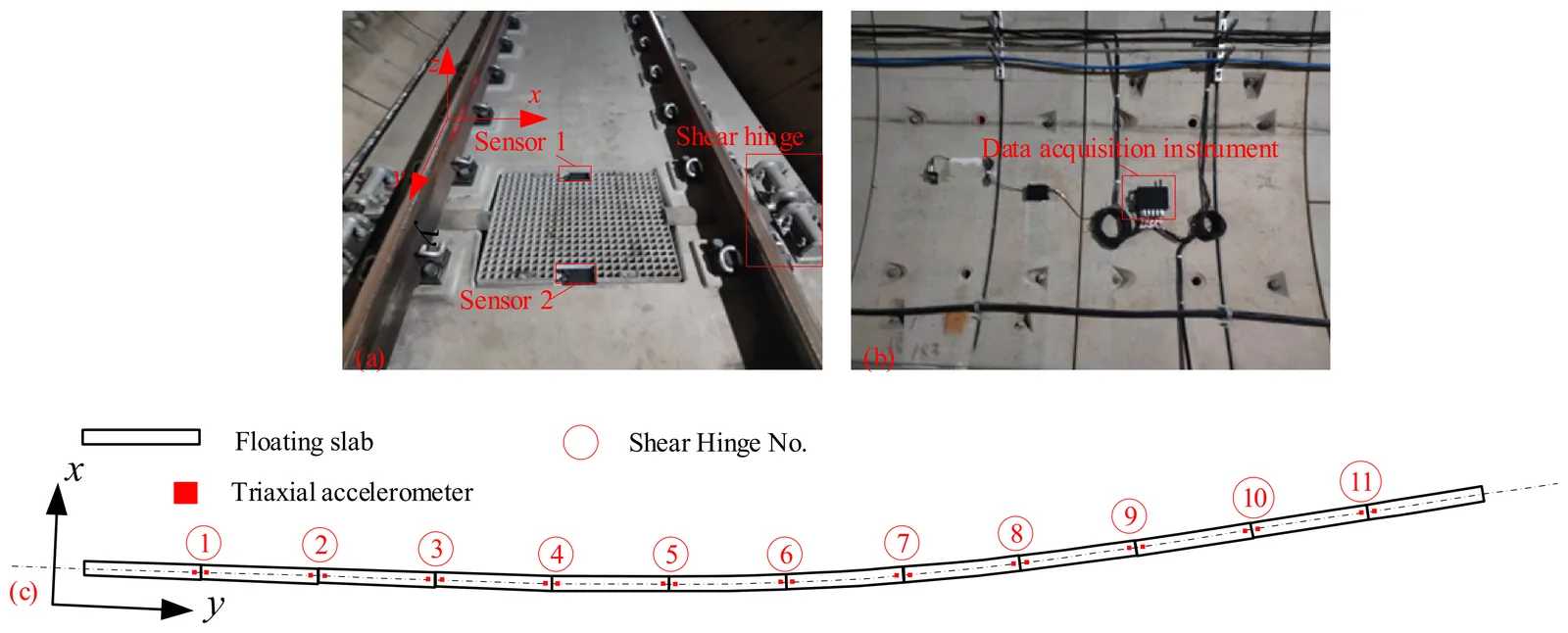
Overall layout of FST line sensor: (**a**) Triaxial accelerometer; (**b**) Data acquisition instrument; (**c**) Overall arrangement of triaxial accelerometer.

The monitoring system consists of two triaxial accelerometers, a GW1605 data acquisition instrument, and a server terminal. The triaxial sensors have a measuring range of ±4 g, a sensitivity of 2048 LSB/g, measurement accuracy of ±20 mg, and a sampling frequency of 500 Hz. The sensors are fixed on the floating slabs at both ends of the shear hinge with screws and epoxy adhesive (Figure 8a), and wired to the data acquisition instrument (Figure 8b). The layout of sensors on a single metro line is shown in Figure 8c.

The experiment covered 11 metro lines with a total of 111 sets of shear hinges, and more than 500 groups of vibration samples were collected by each single sensor. The GW1605 acquisition instrument is equipped with 16 independent acquisition channels, an isolated RS-485 bus, and an ARM real-time computing unit, featuring IP68 dustproof and waterproof protection as well as strong anti-electromagnetic interference capability. When a train passes by, the edge-side algorithm automatically triggers data collection. Multi-channel data are packaged and uploaded to the server to realize long-term continuous acquisition of vibration signals.

All floating slabs here are 25-m cast-in-place slabs, and the shear hinges are top-mounted, as shown in Figure 8c. The x-direction denotes the width of the floating slab, the y-direction denotes its length, and the z-direction is perpendicular to the slab. The operating state of the shear hinge is characterized by combining the three-axis vibration signals from the two vibration sensors.

#### 4.1.2. Dataset Construction

**Figure 9:**
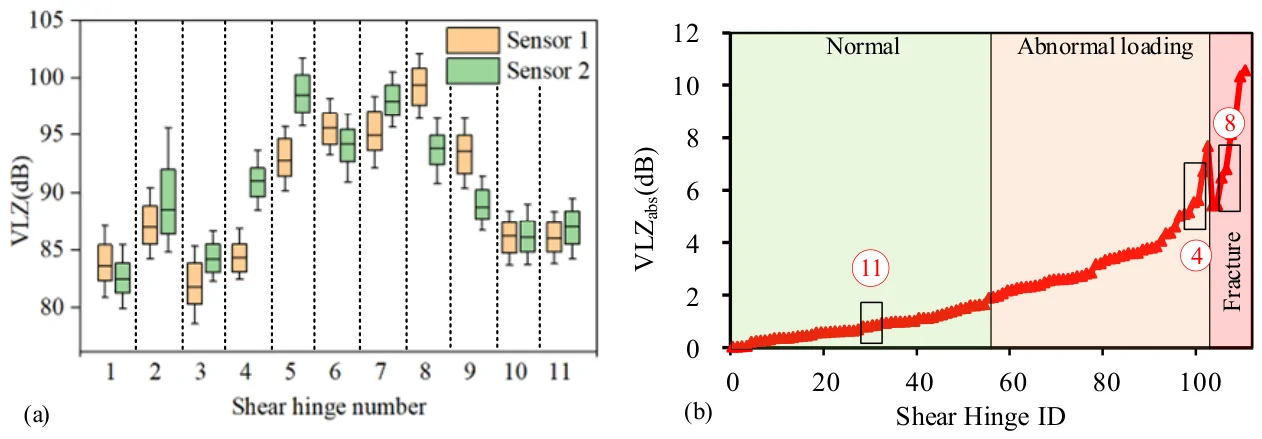
Shear hinge with two-sensor Z-vibration level distribution: (**a**) Single-line shear hinge VLZ; (**b**) All shear hinge VLZ_abs_.

Vibration data of 111 sets of shear hinges from 11 metro lines were collected in this study, and each sample consisted of vibration signals captured by dual triaxial accelerometers during train passage. The Z-weighted Vibration Level (VLZ) was adopted to quantify vibration intensity in engineering applications, as shown in Equation (17):

(17) $$\mathrm{VLZ}=20\mathrm{lg}\frac{a}{a_{0}},$$

where *a* denotes the effective value of Z-weighted vibration acceleration, and *a*_0_ = 10^−6^ m/s^2^ is the internationally unified reference acceleration; the unit of VLZ is dB. The VLZ distribution of the two sensors on a single line is illustrated in Figure 9a. Field inspection verified that shear hinges no. 5 and no. 8 suffered fracture damage, and the VLZ values at damaged positions were significantly higher. It is impossible to judge the component state merely via the amplitude of a single channel, so the VLZ difference between the two sensors must be comprehensively analyzed for state identification.

These shear hinges numbered 4, 8 and 11 correspond to 4, 8 and 11 in Figure 8c and Figure 9a. They are typical representatives of shear hinges under three different fault states. No. 4 is Abnormal-response, No. 8 is Fracture, and No. 11 is Normal.

An absolute mean difference index VLZ_abs_ of VLZ values from dual sensors is defined in Equation (18).

(18) $$\mathrm{VL}Z_{\mathrm{abs}}=\left|\frac{1}{n}{\sum_{i=1}^{n}\mathrm{VLZ}}_{\left(1,i\right)}-\frac{1}{n}{\sum_{i=1}^{n}\mathrm{VLZ}}_{\left(2,i\right)}\right|,$$

To guarantee identification accuracy, the sample quantity *n* shall be greater than 200. Based on the vehicle–track coupling dynamics mechanism and the operation–maintenance engineering experience of urban rail transit floating slab tracks, the judgment criteria for the three service states of shear hinges are given in Equation (19).

(19) $$S=\left\{\begin{array}{ll} \mathrm{Normal}, & {\mathrm{VLZ}}_{\mathrm{abs}}<2\\ \mathrm{Abnormal-response}, & 2\leq {\mathrm{VLZ}}_{\mathrm{abs}}\leq 5,\;{\mathrm{VLZ}}_{1,\mathrm{mean}}<95,\;{\mathrm{VLZ}}_{2,\mathrm{mean}}<95\\ \mathrm{Fracture}, & {\mathrm{VLZ}}_{\mathrm{abs}}\geq 5,\;\left({\mathrm{VLZ}}_{1,\mathrm{mean}}\geq 95\;\mathrm{or}\;{\mathrm{VLZ}}_{2,\mathrm{mean}}\geq 95\right) \end{array}\right.,$$

To guarantee the reliability of ground-truth labels and avoid the randomness of single vibration-level indicators as well as label circular-dependency issues, all samples are independently cross-validated using multi-source field-measured data. All fracture samples are double-verified through on-site open-cover visual inspection and load-transfer performance testing of shear hinges. For instance, shear hinges no. 5 and no. 8 on this line were first identified with structural fracture faults in routine operation-and-maintenance inspections, followed by matching vibration data at the corresponding locations. Hence, their labels are supported by fully independent ground-truth evidence.

For samples with abnormal shear-hinge force conditions, apart from satisfying the VLZ threshold, the pressure measurement records of steel-spring vibration isolators are also matched. Table 1 lists the force-measurement results of isolators corresponding to Figure 9a. The supporting reaction forces of isolators beneath both sides of the floating slab are measured by pressure detectors, so as to verify the causes of abnormal stress such as stiffness degradation and foundation settlement, and ensure that each label corresponds to real structural degradation. Samples of shear hinges in normal condition are collected from locations without fault maintenance records over two consecutive operation–maintenance cycles, where the corresponding isolator pressure tests are qualified. Their normal service conditions are confirmed by the line health operation–maintenance archives.

Based on two-day field-measured VLZ data and on-site inspections, three-category state labeling was performed for 111 shear hinges. The state classification of all shear hinges is illustrated in Figure 9b. The sample distribution conforms to the mechanical degradation law: normal samples account for the largest proportion, followed by samples under abnormal-force conditions, while fracture samples are scarce. A gentle transition exists between normal and abnormal states. After fracture occurs, the difference value first drops and then deteriorates rapidly.

The overall dataset was split into training, validation, and test sets at a ratio of 7:1.5:1.5 based on original physical field-measured samples. The original dataset only contained eight fracture samples, leading to severe class imbalance. To avoid data leakage, multi-intensity Gaussian noise augmentation was adopted to expand fracture samples in the training set for constructing a balanced dataset. The sample partition is presented in Table 2. Multi-level Gaussian noise augmentation was only applied to fracture samples within the already-split training set, yielding 2800 augmented training samples. The validation and test sets entirely retain the original real measured samples without any synthetic augmented samples.

### 4.2. FMTF Processing

Simulated signals and measured signals collected from shear hinges under various working conditions are utilized to verify the effectiveness of the FMTF in enhancing frequency-domain correlation features. Benefiting from full controllability, simulated signals can precisely isolate irrelevant interferences and independently validate the core function of FMTF for strengthening frequency-domain correlation and suppressing random noise. In contrast, field-measured signals of shear hinges with diverse operating states are adopted to examine the practical engineering applicability of the proposed method.

#### 4.2.1. Analysis of FMTF for Simulated Signals

To verify the effectiveness of the FMTF in enhancing frequency-domain correlation characteristics, a simulated signal as shown in Equation (20) is constructed, with a sampling frequency *f_s_* = 20 Hz.

**Figure 10:**
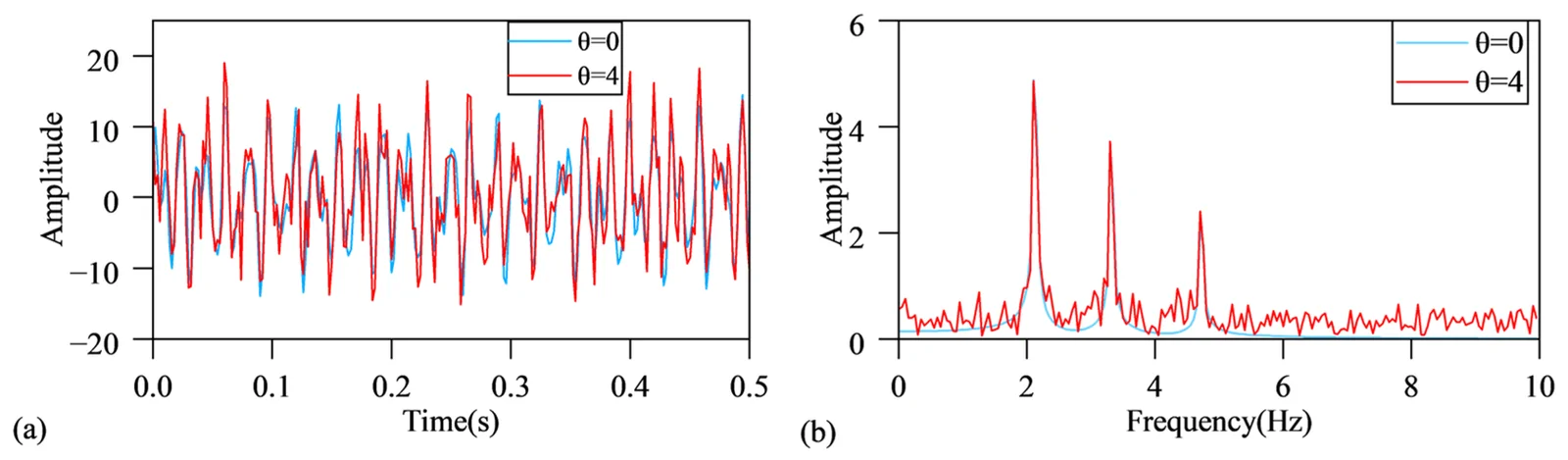
Simulation signal and corresponding frequency spectrum: (**a**) Time-domain waveform; (**b**) Frequency spectrum.

*x*(*t*) = 7 × sin(2.123 × 2 × *πt*) + 5 × cos(3.321 × 2 × *πt*) + 3 × sin(4.723 × 2 × *πt*) + *θ* × *ξ*(*t*), (20)

where *ξ*(*t*) is standard normal random noise, *ξ*(*t*)∼𝒩(0,1), and θ is the noise intensity. When *θ* = 4, the time-domain waveform and frequency spectrum of the signal are shown in Figure 10.

**Figure 11:**
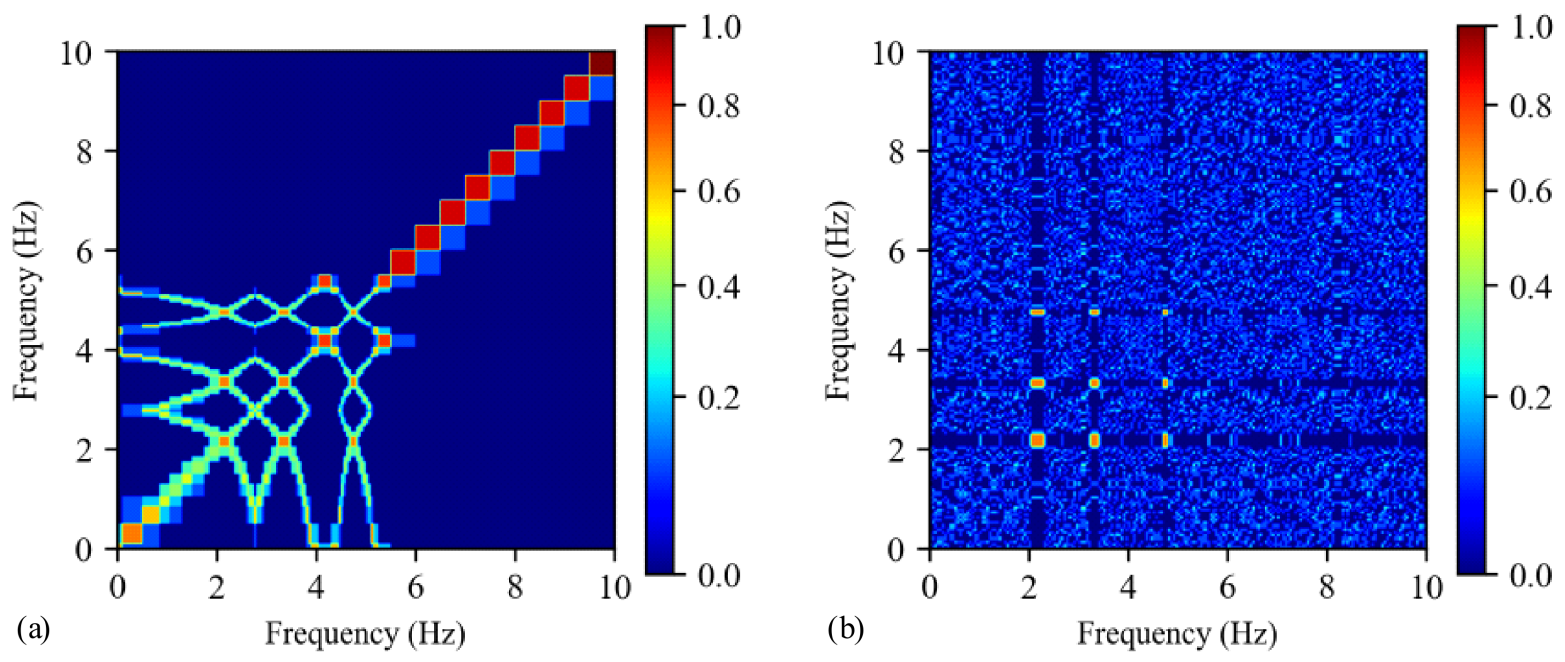
FMTF of Simulated Signals with Various Noises: (**a**) *θ* = 0; (**b**) *θ* = 4.

Using equal-frequency division with 20 quantiles, the FMTF for noise levels *θ* = 0 and *θ* = 4 are calculated, as shown in Figure 11. When *θ* = 0, the main diagonal exhibits high probability, and there are clear, regular bright spots in the off-diagonal regions, corresponding to the correlations between different frequency components of the signal; the background is clean, free from random noise interference, directly reflecting the inherent spectral correlation characteristics of the signal. When *θ* = 4, the background is covered by a large amount of noise (blue scattered points), and the frequency structure of the original signal is severely submerged; however, the core pattern is still preserved—the high-probability feature of the main diagonal and the key bright spots corresponding to the original signal frequencies remain identifiable against the noisy background.

Thus, the FMTF can highlight the characteristics of spectral distribution, enhance the correlation between different frequencies, and exhibit strong noise immunity. When the state of the shear hinge changes, the variation in its spectral distribution becomes more complex. However, the properties of the FMTF enable it to capture and process such complex spectral variations more accurately and efficiently, fully leveraging its advantages in spectral feature analysis.

#### 4.2.2. FMTF Analysis

1. Spectrum Analysis

**Figure 12:**
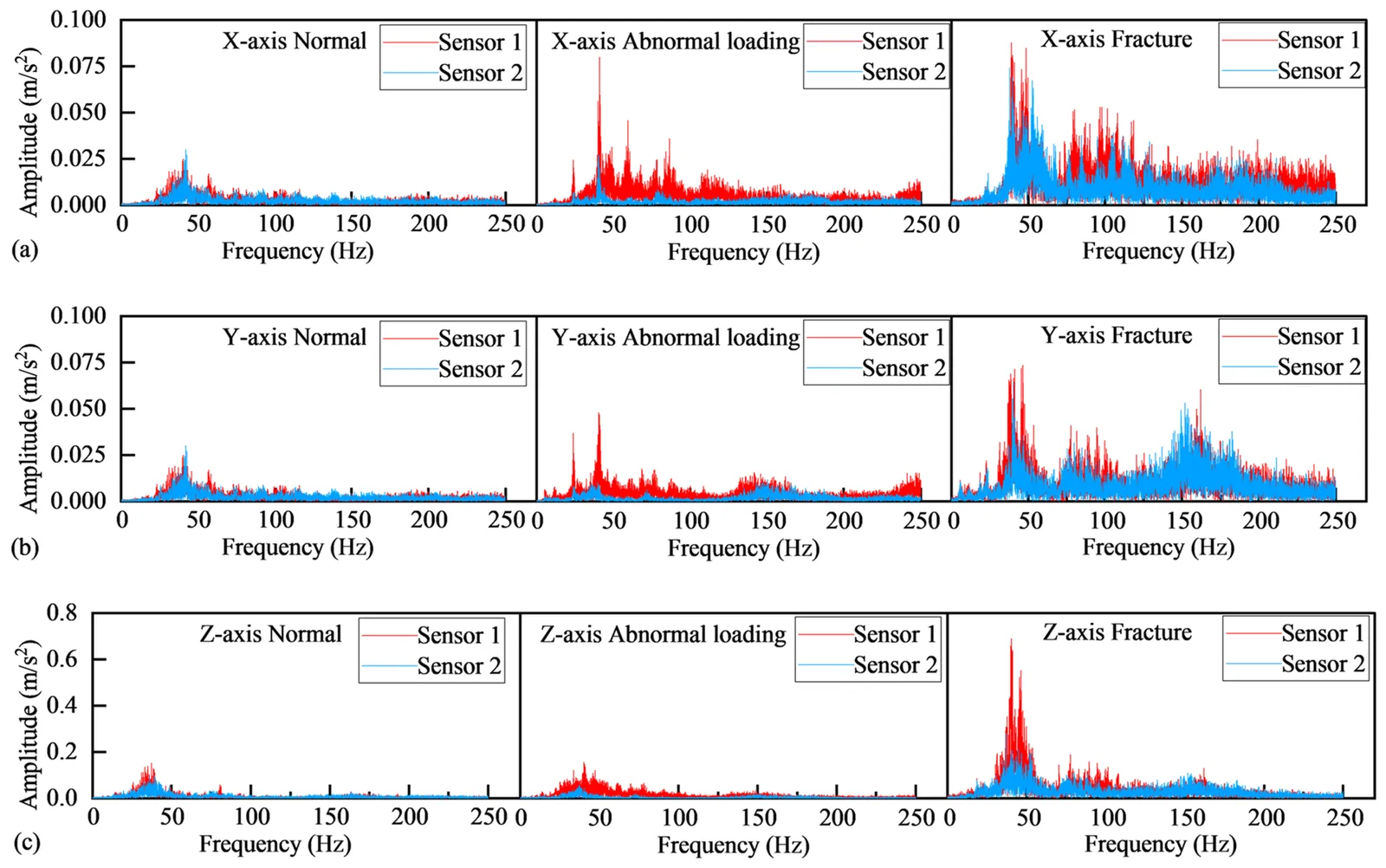
Shear hinge spectra in different states: (**a**) X-axis; (**b**) Y-axis; (**c**) Z-axis.

Each state sample of the shear hinge contains triaxial vibration signals collected from two sensors. The raw vibration signals are processed by mean removal, followed by Fourier transform to analyze the triaxial vibration spectra of floating slabs under three service states, as illustrated in Figure 12. The dynamic evolution mechanism of shear hinges is interpreted based on the FST mathematical model proposed in reference [5].

When the shear hinge is in normal condition, the floating slabs on both sides possess identical support stiffness, fastener damping, and vibration isolation performance. System vibration is dominated by global low-order modes within 0–50 Hz. Due to structural damping and energy dissipation, high-frequency modes above 50 Hz are barely excited, which matches the spectral characteristics of the no. 11 intact shear hinge in Figure 12.

Excessive load on the shear hinge originates from foundation settlement or degraded isolator performance, which reduces the support stiffness of one floating slab and differentiates the dynamic behaviors of the two slabs (corresponding to the no. 4 shear hinge in Figure 8). The vibration amplitude rises in the low-frequency band of 0–50 Hz. Meanwhile, system stiffness nonlinearity generates abundant vibration components ranging from 50 to 200 Hz, leading to remarkable spectral discrepancy between signals of the two sensors.

Once the shear hinge fractures, the continuity of adjacent floating slabs is destroyed, and the multi-span continuous beam on an elastic foundation is decoupled into several independent single-span beams (corresponding to the fractured no. 8 shear hinge in Figure 6). The transverse and longitudinal shear stiffness at the fracture position drops sharply to zero, the elastic constraints at slab ends fail, and the vibration transmission path between slabs is cut off.

X-axis vibration is mainly excited by centrifugal forces on curved tracks and wheel–rail lateral forces. Fracture eliminates lateral constraints, aggravates lateral deformation of floating slabs and excites high-order shear modes, producing new spectral components from 50 Hz to 200 Hz. Y-axis vibration is dominated by longitudinal train impact forces and wheel–rail creep forces. The loss of longitudinal constraints after fracture induces relative displacement between adjacent slabs and excites torsional modes, generating new spectral peaks within 50–150 Hz. Z-axis vibration is determined by vertical train loads and vertical bending stiffness of steel springs. After fracture, vertical loads concentrate on a single floating slab, activating high-order bending modes and causing continuous amplitude growth across the full frequency band.

According to the above analysis, the identification of shear hinge operating states relies on specific frequency bands extracted from dual-end vibration spectra rather than single frequency components. This unique property limits the applicability of conventional fault diagnosis algorithms developed for rotating machinery. Therefore, the frequency-band differential features extracted from vibration signals at both ends of the shear hinge should be prioritized for condition monitoring.

2. MTF Analysis

**Figure 13:**
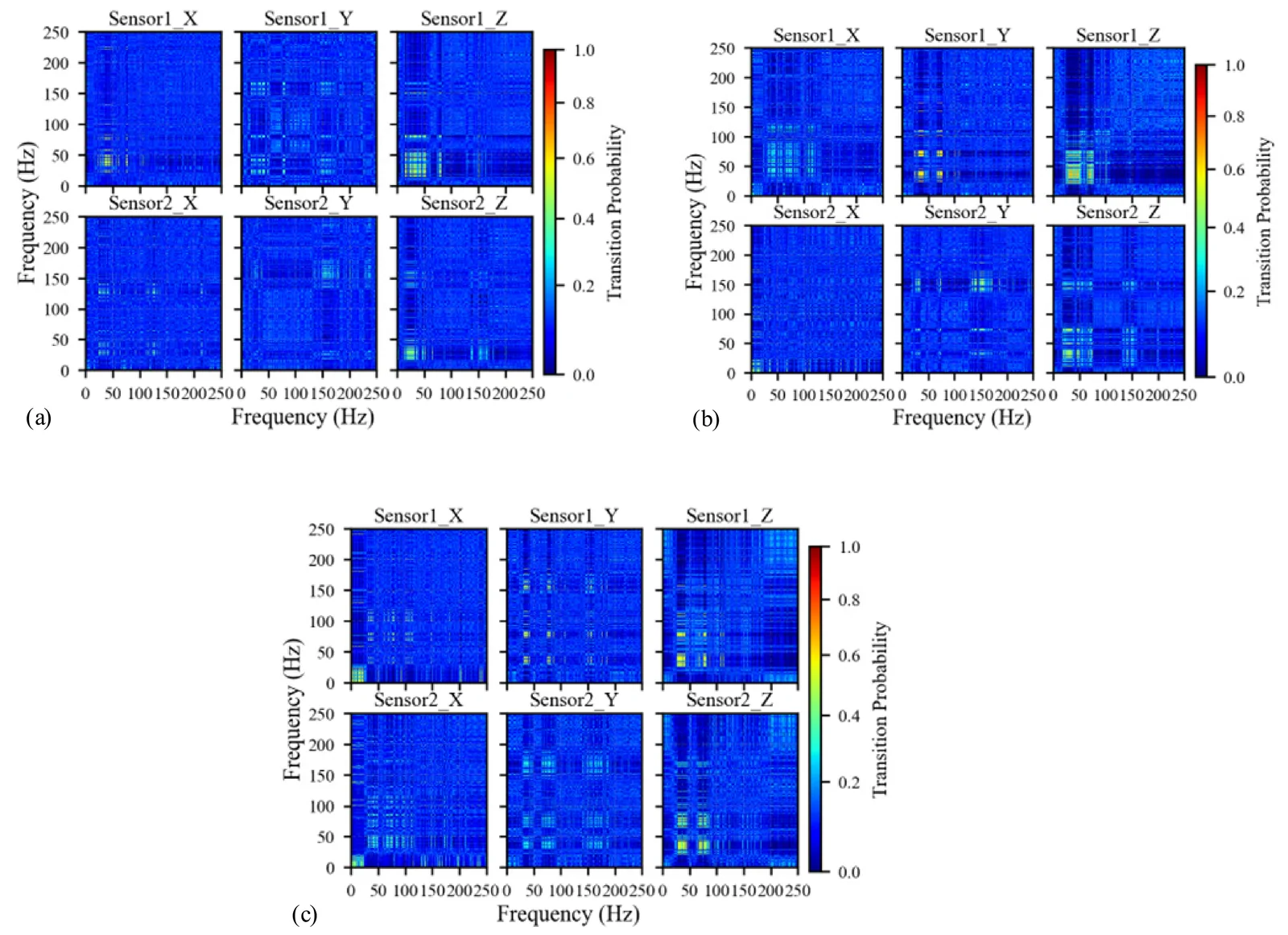
FMTF of shear hinge under different conditions: (**a**) Normal; (**b**) Abnormal-response; (**c**) Fracture.

After Fourier transform, a spectrum of 0–250 Hz with a length of 5000 was obtained. To avoid an excessively large size of the two-dimensional FMTF, the spectrum was uniformly sampled with a step size of 20, yielding a spectrum of length 250. The quantile of MTF was set to 10 with equal-frequency division, and the image size was 250 × 250. The FMTF of the three components from the two sensors were obtained, as shown in Figure 13, covering three working states. With the deterioration of the shear hinge condition, the differences between different components of the two sensors increase significantly.

When the shear hinge is in normal condition, as shown in Figure 13a, the FMTF of the two sensors are similar. The high-probability regions are concentrated and small in range, with most areas appearing blue, indicating stable and concentrated transition patterns between frequencies without obvious fluctuations. When the shear hinge suffers from abnormal force, as shown in Figure 13b, the high-probability regions begin to disperse, yet the overall distribution remains dominated by blue. This suggests that the transition patterns between frequencies experience fluctuations and reduced stability, but no severe disorder occurs. When the shear hinge is fractured, as shown in Figure 13c, the high-probability regions increase significantly and expand in range, with even orange regions appearing. The high-probability distributions of each component become more scattered, indicating severe disorder in the transition patterns between frequencies and enlarged differences in transition probabilities across different frequencies, which is a typical fault characteristic.

The transition probability distribution between frequencies clearly distinguishes the state changes of the shear hinge among normal, abnormally loaded, and fractured conditions, and can even identify potential anomalies adjacent to fracture. The directional differences captured by the sensors can thus serve as a valid basis for shear hinge condition identification.

### 4.3. PCNN-RWKV Model Validation

#### 4.3.1. Model Training Parameter Settings

To ensure the fairness of model evaluation, all experiments were conducted under the same experimental environment. The model was built using PyTorch 1.12.1, and an NVIDIA RTX 3060 was used as the hardware platform to evaluate the proposed method. Cross-entropy loss was adopted as the model loss function. The Adam optimizer was used with a learning rate of 0.0001. Due to limited computing resources, the batch size was set to 4. To ensure sufficient model training, the maximum number of epochs was set to 20, with other conditions kept at default.

To comprehensively evaluate the overall performance of the proposed model, we adopt metrics from two dimensions: classification capability and deployment efficiency. In terms of classification performance, Accuracy (Acc) measures the overall recognition accuracy of the model. Precision (Pre) represents the model’s ability to avoid false predictions, while Recall (Rec) reflects its capability to detect all actual faults. As the harmonic mean of precision and recall, F1 Score (F1) is used to synthetically assess the classification performance. For deployment efficiency, GFLOPs indicates the computational complexity, and Parameters (Params) characterizes the memory and storage overhead. Frames Per Second (FPS) and Latency evaluate the inference speed and single-sample processing time, respectively, which further verify the applicability of the model for real-time monitoring tasks.

#### 4.3.2. Study on Optimal Structural Parameters of PCNN-RWKV Model

According to the characteristics of shear hinge state signals, the number *M* of parallel CNNs in PCNN-RWKV is set to 6. Factors affecting the performance of PCNN-RWKV include the branch number *F* of ELAN-F in PCNN and the number *d* of RWKV nodes. The influence of these factors on model performance is determined through single-factor experiments in the following sections. The dataset is divided into training, validation, and test sets following a 7:1.5:1.5 ratio. Parameters F and d are screened purely according to validation-set performance. The test set is excluded from model training, hyperparameter tuning, and network architecture selection throughout the whole process.

1. The Impact of PCNN Parameters on Model Performance

**Figure 14:**
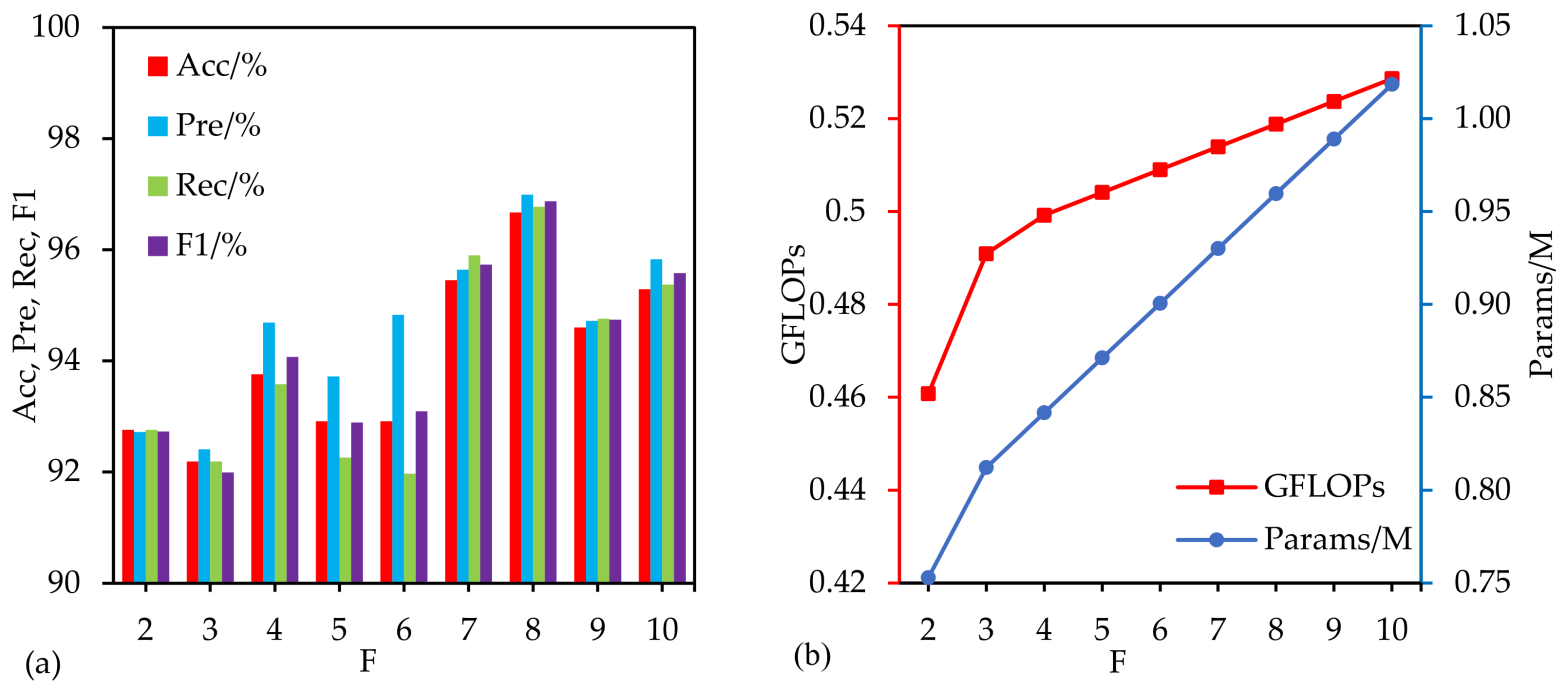
Influence of multi-scale number F on PCNN performance: (**a**) Impact of *F* on Classification Performance; (**b**) Scalability of GFLOPs and Parameters vs. *F*.

The core parameter affecting PCNN performance is the multi-scale number *F* of ELAN-F. The influence of *F* on PCNN performance was verified when *F* was set to [2, 3, …, 10]. On the validation set, the mean value of five repeated experiments is adopted as the model evaluation metric for hyperparameter selection, as shown in Figure 14.

As shown in Figure 12, as *F* increases from 2 to 10, the model’s accuracy, precision, recall, and F1-score show an overall upward trend. All indicators reach their peak at *F* = 8, indicating that increasing *F* effectively improves the model’s ability to extract and identify shear hinge state features. As *F* increases, the model’s GFLOPs rise from 0.49 to 0.52, and Params grow slowly from 0.75 to 1.02. This shows that increasing the multi-scale number of ELAN-F mainly raises the model’s parameter count rather than its computational complexity. Overall, when *F* = 8, the core metrics including classification accuracy, recall, and F1-score of the model on the validation set all reach their peaks, yielding the optimal overall recognition performance. Although *F* = 7 brings slight advantages in computational cost and parameter quantity, it suffers a 1.22% drop in recognition accuracy. Therefore, *F* = 8 with the best overall performance is selected as the final model parameter. For edge-end deployment scenarios with limited computing resources, *F* = 7 can serve as a compromise scheme that balances accuracy and operational efficiency. In the subsequent experiments, F is fixed to 8 for the model.

2. Effect of RWKV Parameters on Model Performance

**Figure 15:**
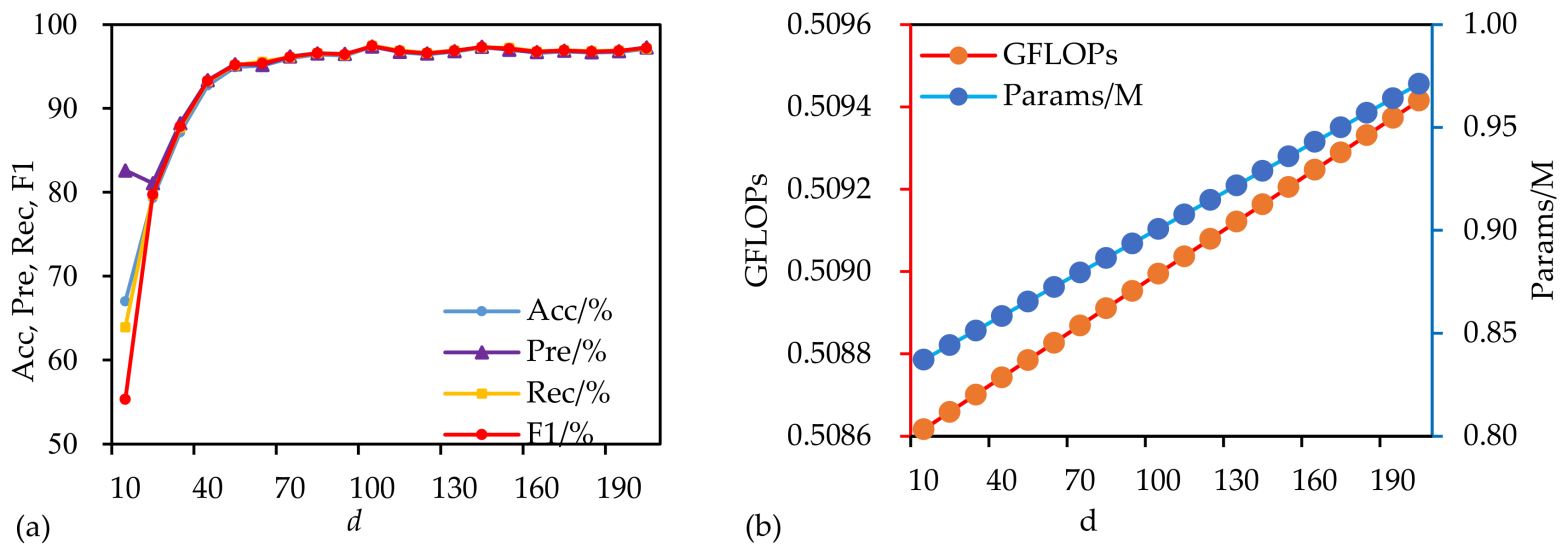
The influence of the number of hidden layer nodes on the performance of PCNN-RWKV: (**a**) Impact of RWKV Hidden Dimension on Classification Performance; (**b**) Scalability of GFLOPs and Params vs. RWKV Hidden Dimension.

The core parameter affecting RWKV performance is the number of hidden layer nodes *d*. By gradually increasing *d* from 10 to 200 with a step size of 10, its influence on the performance of PCNN-RWKV was verified. On the validation set, the average value of five experiments was taken as the final evaluation result of the model, as shown in Figure 15.

As can be seen from Figure 15, as the number of hidden layer nodes of RWKV increases to 100, the model’s accuracy, precision, recall rate, and F1-score all rise rapidly, indicating that increasing the number of hidden layer nodes can effectively enhance the model’s ability to model cross-channel temporal dependencies. When the number of nodes exceeds 100, all performance indicators tend to saturate, with the improvement becoming gradual and approaching a peak of nearly 98%, suggesting that the model’s feature representation capability is close to optimal at this point. Meanwhile, as the number of hidden layer nodes of RWKV increases, the model’s GFLOPs and Params grow approximately linearly: the number of parameters rises from about 0.85 M to 0.95 M, and the computational load increases from approximately 0.5086 to 0.5095, which directly raises the model’s complexity and computational overhead. Overall, under the experimental conditions of this study, setting the number of hidden layer nodes of RWKV to around 100 represents an ideal balance between performance and efficiency, as it enables the model to achieve near-peak performance while effectively controlling computational costs.

It can be concluded from the above analysis that on the shear-hinge state dataset, the PCNN-RWKV shear-hinge condition recognition model achieves optimal performance when the branch number *F* of ELAN-F in PCNN is set to 8 and the number of RWKV nodes is set to 100.

#### 4.3.3. Ablation Study

**Figure 16:**
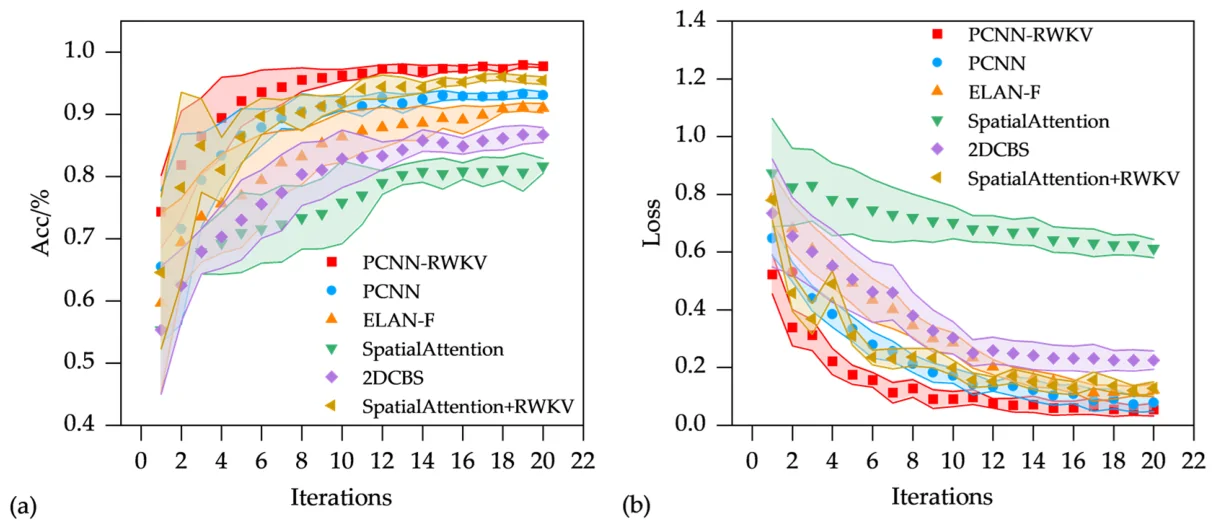
Accuracy and loss convergence curves of different module combination models on the validation set: (**a**) Variation of validation accuracy with the number of iterations; (**b**) Variation of validation loss with the number of iterations.

PCNN-RWKV consists of two core modules: PCNN and RWKV. The PCNN module further includes Spatial Attention and ELAN-F. To further clarify the influence of these modules on model performance, an ablation study is conducted on the test set of the shear hinge state dataset to demonstrate their contribution to the model. On the validation set of the shear hinge state dataset, the curves of loss value and accuracy versus the number of iterations are shown in Figure 16.

As shown in Figure 16a, the accuracy of all models gradually increases and tends to converge with the number of iterations. Both the PCNN and PCNN-RWKV models can achieve an accuracy of 95%. Among them, PCNN-RWKV converges the fastest and exhibits the best performance. The model containing only the spatial attention module achieves the lowest accuracy and converges the slowest. The introduction of ELAN-F and RWKV can significantly improve the classification accuracy of the model, and the synergistic effect of multiple modules is superior to that of a single module. From the loss curves in Figure 16b, it can be seen that ELAN-F and RWKV can effectively reduce the model loss and improve the convergence speed and stability of the model.

To comprehensively evaluate the overall performance of the model, the mean values of five independent experiments on the test set of the shear hinge state dataset are taken as the final evaluation results, as shown in Table 3.

As presented in Table 3, when only the Spatial Attention module is retained, the model achieves a precision of merely 81.49% and an F1-score of 81.40%. This indicates that the Spatial Attention module can effectively boost the model’s attention to fault-related FMTF. When 2DCBS is stacked in two layers, although the accuracy is improved, the computational overhead is large. ELAN-F alone can achieve better performance and lower computational complexity, which is better than ordinary stacked convolution. After incorporating the ELAN-F module, the precision surges to 94.47% with an F1-score of 94.21%, which verifies that PCNN possesses powerful multi-scale feature extraction capability. Further embedding the RWKV module improves the precision by 3.42%, delivering additional performance gains on top of the already high baseline accuracy, demonstrating that RWKV effectively strengthens the model’s ability to capture cross-channel features.

**Figure 17:**
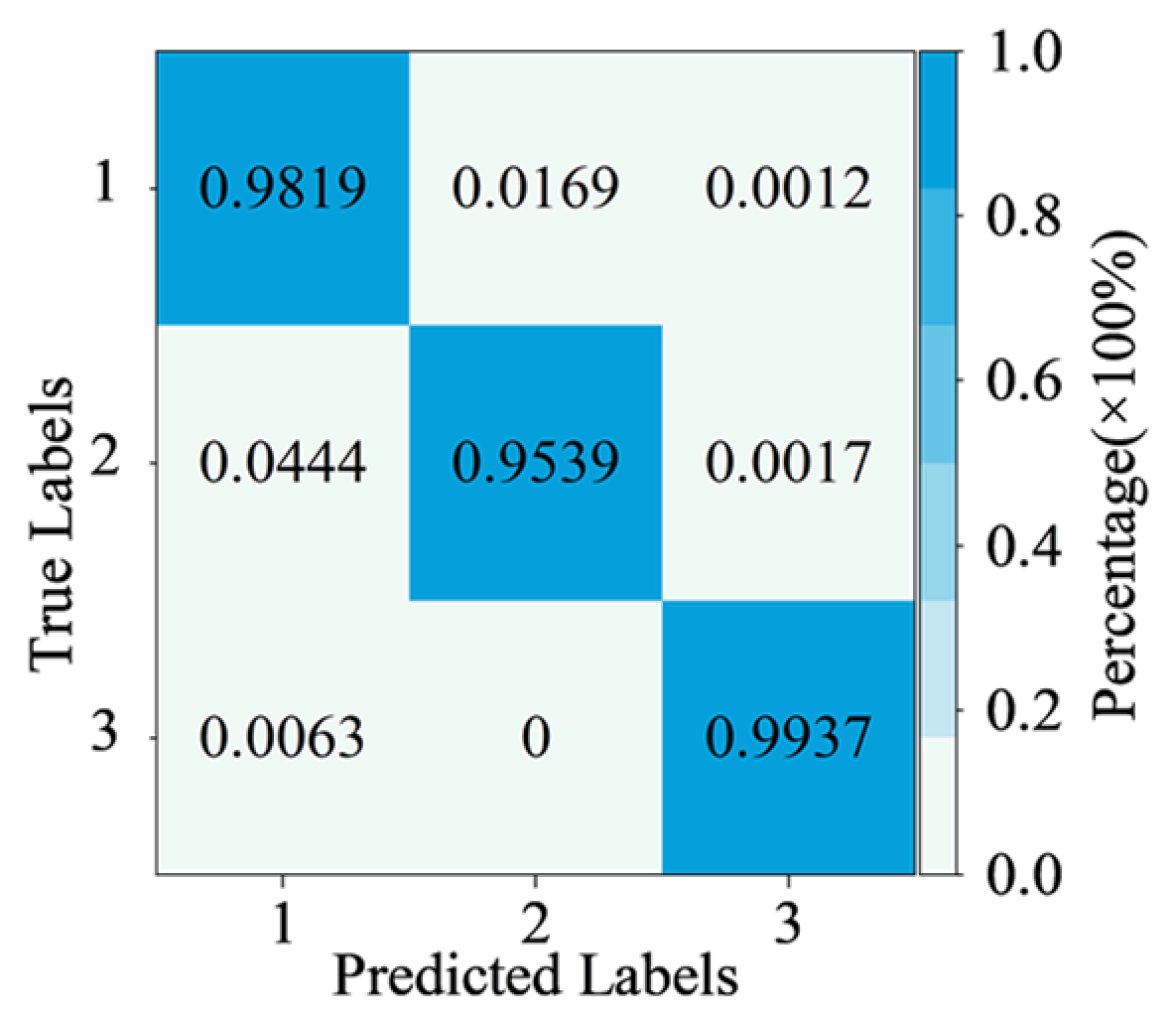
PCNN-RWKV Confusion Matrix.

The three-class confusion matrix of PCNN-RWKV is illustrated in Figure 17.

As shown in Figure 17, the accuracy of the model on all three categories exceeds 95%, and the recognition accuracy for shear hinge fracture is as high as 99.37%. The errors are mainly between the normal shear hinge state and the abnormal force state of the shear hinge, both of which are less than 5%, indicating that the model achieves high recognition accuracy on the three states.

**Figure 18:**
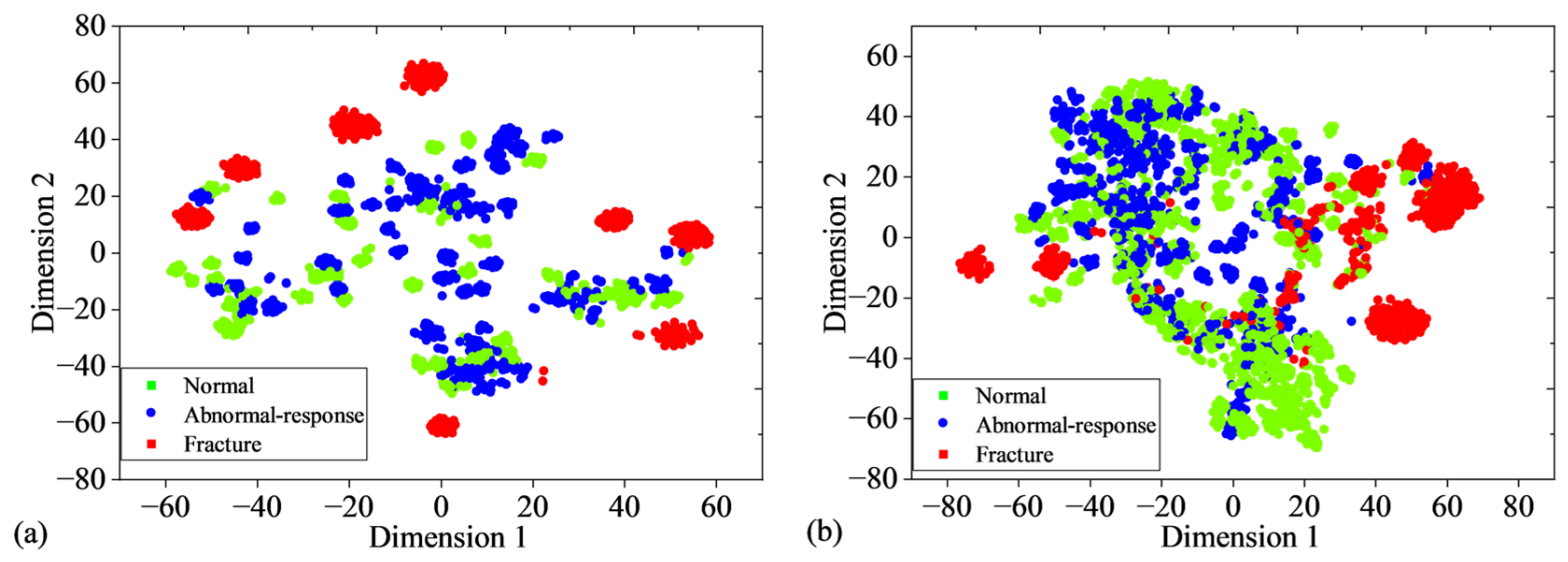

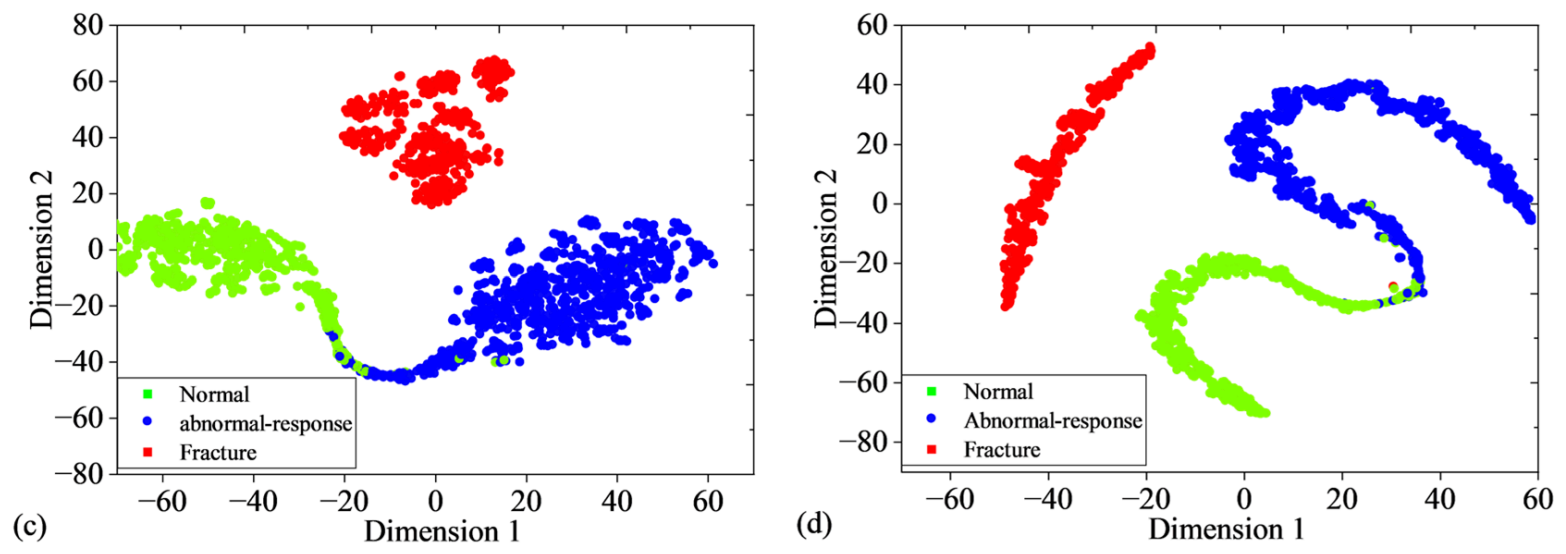
T-SNE distribution of features from PCNN-RWKV modules: (**a**) FMTF features; (**b**) PCNN features; (**c**) RWKV features; (**d**) Full connection layer features.

The t-distributed stochastic neighbor embedding (T-SNE) algorithm is employed to reduce the features extracted by each sub-module of PCNN-RWKV to a two-dimensional space, and the results are shown in Figure 18.

As can be seen from Figure 18a, after FMTF processing, different shear hinges exhibit obvious individual differences, while the three states of the shear hinges are completely randomly mixed together and difficult to distinguish. After feature extraction by PCNN, as shown in Figure 18b, shear hinges of the same category gradually cluster together, but it is still challenging to identify the shear hinge state solely based on the multi-component FMTF. By leveraging RWKV to extract dependency features across different components, the features can be clearly divided into three distinct clusters, as illustrated in Figure 18c. When mapped to labels via the full connection layer, the shear hinge states can be easily identified, as presented in Figure 18d. Among them, the shear hinge fracture state is significantly different from the other two states, with basically no overlap.

There is an obvious state transition between the normal state and the abnormal-response state of shear hinges, leading to partial overlap between corresponding labels. This phenomenon is fully consistent with the gradual transition zone illustrated in Figure 9. The vibration characteristics of shear hinges evolve continuously and slowly, instead of changing abruptly between normal and abnormal operating conditions, thus forming an inherent ambiguous transition region between the two categories. The results demonstrate that the features extracted by PCNN-RWKV can effectively reflect the actual working conditions of shear hinges. To quantitatively evaluate the features extracted by different modules, we employed the Silhouette score, Davies–Bouldin index, and k-NN classification accuracy, as shown in Table 4.

As can be seen from Table 4, the original features extracted by FMTF and PCNN do not exhibit effective class separability. Although their k-NN accuracies are acceptable, the Silhouette scores and Davies–Bouldin indices are poor, indicating severe overlap in the feature distribution, and the apparent separation in the visualization is misleading. The RWKV fusion module significantly improves the clustering quality of the features: the Silhouette score increases from 0.03 to 0.87, while the Davies–Bouldin index decreases from 8.26 to 0.18, proving that it is the most critical feature enhancement module in the model. Meanwhile, the features at the final output layer maintain high clustering quality while achieving the highest k-NN accuracy, striking an optimal balance between clustering quality and classification performance.

In summary, FMTF can better characterize the state variation of shear hinges and exhibits favorable interpretability. Each module of PCNN-RWKV contributes to improving the overall performance of the model, showing a synergistic optimization effect. Among them, ELAN-F and RWKV serve as core modules for performance enhancement; RWKV further optimizes model performance with low computational and parameter overhead. Experimental results verify the necessity and efficiency of each module, which can effectively identify the three states of shear hinges and provide reliable technical support for rail operation and maintenance.

#### 4.3.4. Influence of Spectral Resolution on Model Performance

**Figure 19:**
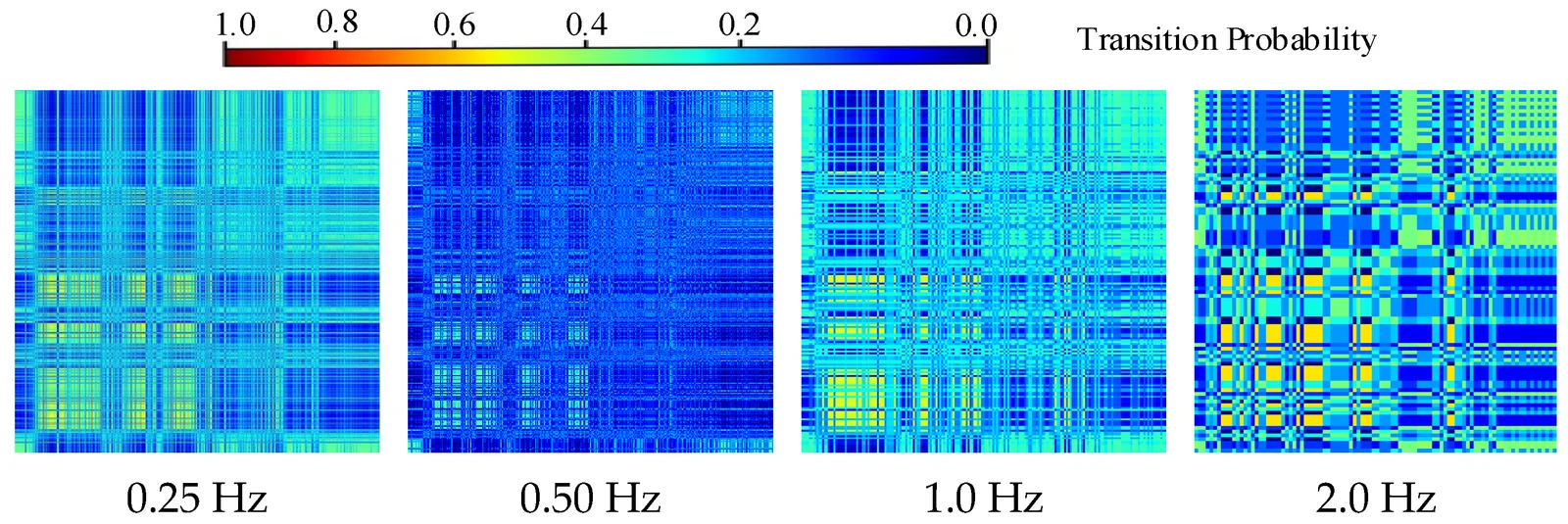
FMTF with different spectral resolution.

Spectral resolution determines the degree of feature preservation of FMTF. The spectral *step* is set to [5, 10, 20, 40], corresponding to frequency-domain resolutions of 0.25 Hz, 0.50 Hz, 1.0 Hz, and 2.0 Hz, respectively. The obtained FMTF results are shown in Figure 19.

**Figure 20:**
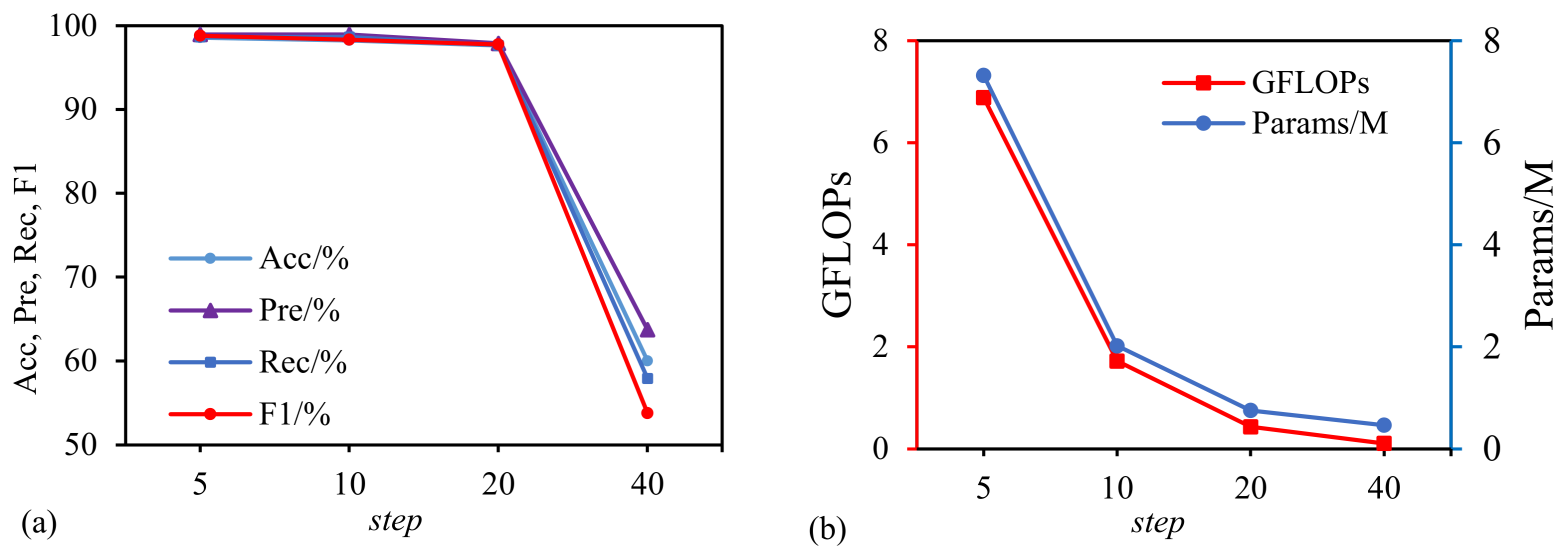
Recognition performance of PCNN-RWKV under different spectral resolutions: (**a**) Impact of *step* on Classification Performance; (**b**) Scalability of GFLOPs and Params vs. *step*.

It can be observed from Figure 19 that the features of FMTF become increasingly blurred as the resolution decreases. Next, the model is retrained on shear-hinge-state datasets with different frequency-domain resolutions. The model performance is tested under each respective resolution condition, and the test results are presented in Figure 20.

It can be observed from Figure 20 that the recognition performance of PCNN-RWKV decreases monotonically as the FMTF resolution declines. When the resolution is sufficiently high, the recognition accuracy of the model can reach 98%. Nevertheless, excessively high resolution leads to difficulties in data storage and model training. When *step* > 20, the recognition performance of the model degrades rapidly and the identification accuracy becomes inadequate. Considering both recognition performance and model complexity comprehensively, the FMTF with a *step* size of 20, corresponding to a resolution of 1 Hz, is selected for model performance validation.

#### 4.3.5. Influence of Signal Encoding Methods on Model Recognition Performance

To verify the effectiveness of FMTF, comparative experiments are carried out with classic two-dimensional encoding methods including one-dimensional spectrum (FFT), time-domain Gramian angular field (TGAF), time-domain Markov transition field (TMTF), STFT, CWT, and frequency-domain Gramian angular field (FGAF). In all experiments, the sampling frequency is uniformly set to 500 Hz, and raw vibration signals with an interception length of *N* = 4000 are adopted. The output feature map size of all six encoding methods is fixed at *S*^2^ = 250 × 250. For CWT, the Morlet wavelet with 200 scales is convolved with the full-length signal. For STFT, the window length is L = 256 and the number of frame overlap points is 220, generating a total of 105 short-time Fourier transform frames. Both FGAF and FMTF perform only one 4000-point FFT to construct the 250 × 250 feature matrix. Since FFT outputs one-dimensional features, the feature-extraction module of PCNN-RWKV is replaced with a convolution module for one-dimensional features with 250 feature points to guarantee experimental fairness. On the shear-hinge condition test set, PCNN-RWKV with identical hyperparameters is employed, and the mean value of five repeated experiments is taken as the final evaluation result, as shown in Table 5.

As can be seen from Table 5, FMTF achieves comprehensive recognition performance comparable to the classic STFT method, with an accuracy of 97.62%, which is 0.58% higher than STFT. Its precision, recall, and F1-score all remain steadily above 97.6%, substantially outperforming TMTF, TGAF, and CWT. Furthermore, a paired-sample *t*-test is conducted on five groups of test accuracy results of FMTF and STFT, yielding *p* = 0.021 < 0.05. It demonstrates that the performance improvement of FMTF over STFT is statistically significant and not an accidental outcome caused by random initialization. Although FFT has the lowest time complexity and yields higher accuracy than TMTF and TGAF, its overall performance is inferior to FMTF. Under identical computational cost, FMTF is capable of mining nonlinear coupled features in the frequency domain, and its fault recognition accuracy is 3.08% higher than that of FGAF. Nevertheless, the state variation of shear hinges in floating slab tracks is extremely slow. While STFT can extract time-varying and frequency-varying characteristics of signals, it introduces redundant information for shear-hinge tasks, resulting in poorer frequency-domain interpretability compared with FMTF. By contrast, FMTF focuses on frequency-domain variations and possesses lower time complexity, which makes it more suitable for shear-hinge condition recognition.

#### 4.3.6. Effects of FMTF Hyperparameters on Model Performance

**Figure 21:**
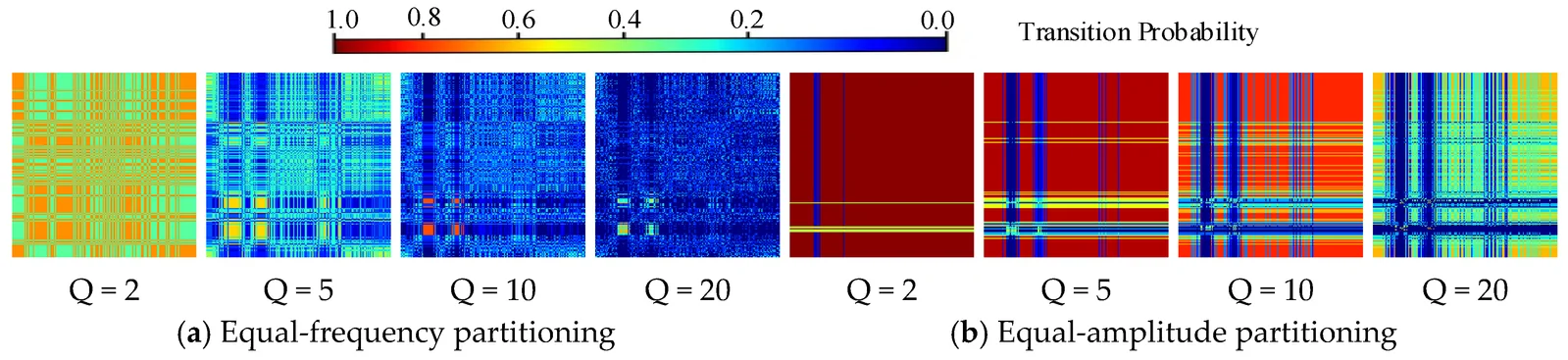
Z-axis FMTF of fractured shear hinges with varied partitioning schemes.

Factors affecting FMTF transformation include partitioning strategy and quantile number. Partitioning strategies include equal-frequency partitioning and equal-amplitude partitioning. The resulting FMTFs for quantile *Q* ranging from 2 to 10 are shown in Figure 21.

As shown in Figure 21a, under the equal-frequency partitioning strategy, frequency-related regions in the FMTF form rectangular blocks. As the quantile number increases, the resolution of different regions gradually improves, but the amplitude information of the spectrum is lost. Low-energy spectral components form color-similar mosaic patterns, thereby highlighting high-energy frequency components and suppressing the influence of noise on critical frequency bands. As shown in Figure 21b, under the equal-amplitude partitioning strategy, frequencies with the same amplitude are directly assigned the same color, and low-energy spectra are compressed into a single color. This achieves more significant noise suppression but also causes the loss of correlation information between different frequencies.

**Figure 22:**
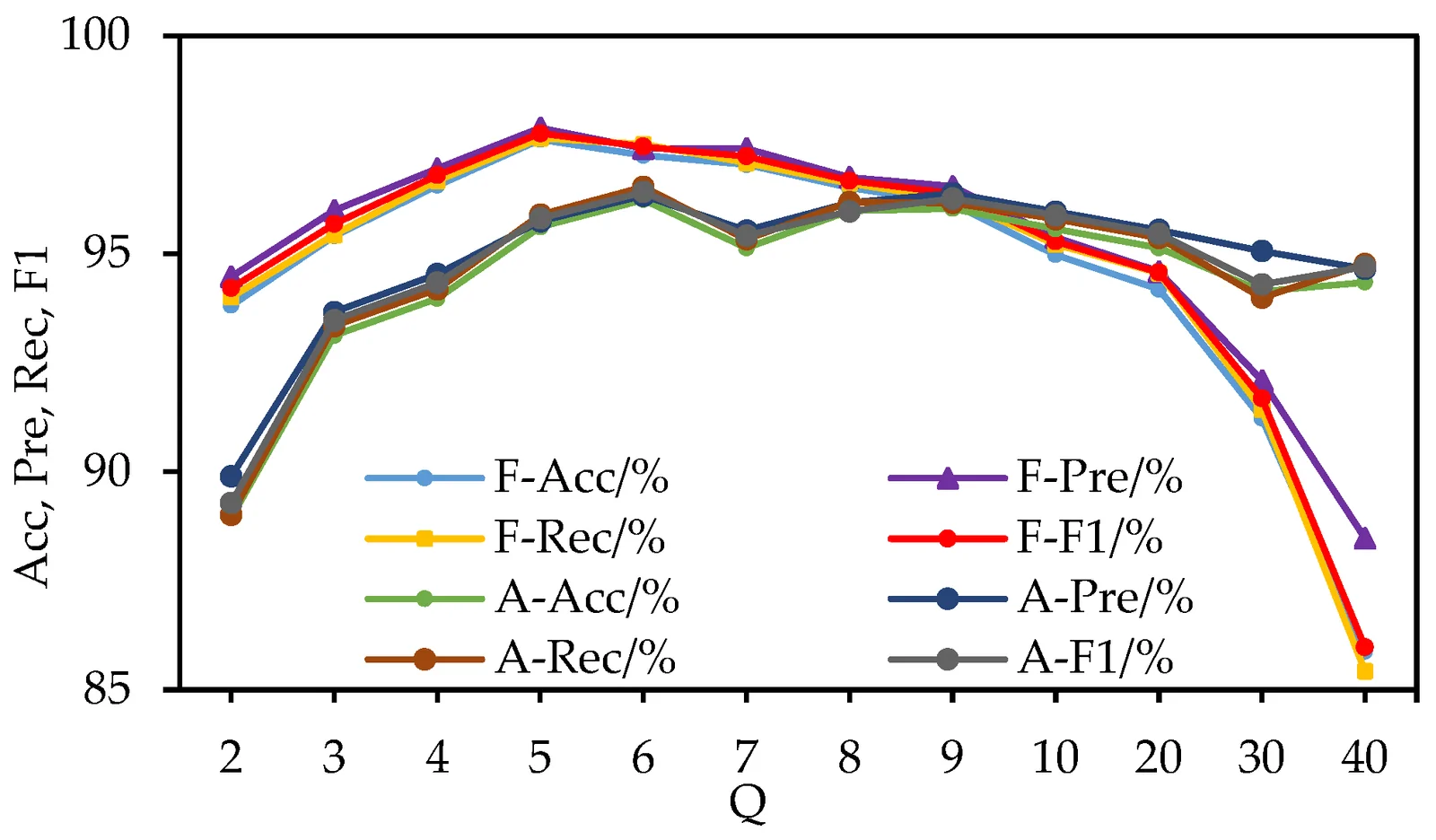
The impact of FMTF partitioning strategy and quantile on recognition performance.

Subsequently, experiments are conducted on the shear hinge state dataset using the PCNN-RWKV model with identical parameters. The mean results of five repeated trials are taken as the final evaluation metrics, as shown in Figure 22. Here, F-xxx denotes equal-frequency partitioning, while A-xxx denotes equal-amplitude partitioning.

As shown in Figure 22, for both partitioning strategies, all evaluation metrics increase rapidly with quantile *Q*, reach a peak, and then slowly decline. Throughout the range, the metrics for equal-frequency partitioning are consistently higher than those for equal-amplitude partitioning, demonstrating that FMTF features constructed by equal-frequency partitioning are better suited to the proposed model and yield superior shear hinge state recognition performance. The optimal hyperparameter configuration for FMTF is *Q* = 5. When *Q* < 5, the partitioning intervals are too coarse, leading to insufficient feature granularity and loss of details, which degrades recognition accuracy. When *Q* > 6, excessive partitioning introduces redundant noise features and weakens correlation information between different frequencies, resulting in reduced model generalization and lower recognition precision.

#### 4.3.7. Effect of Noise on Model Performance

To verify the robustness of the model under noisy conditions, robustness tests are carried out by adding Gaussian noise and impulse-shock noise to the original signals.

1. Influence of Gaussian Noise on Model Performance

**Figure 23:**
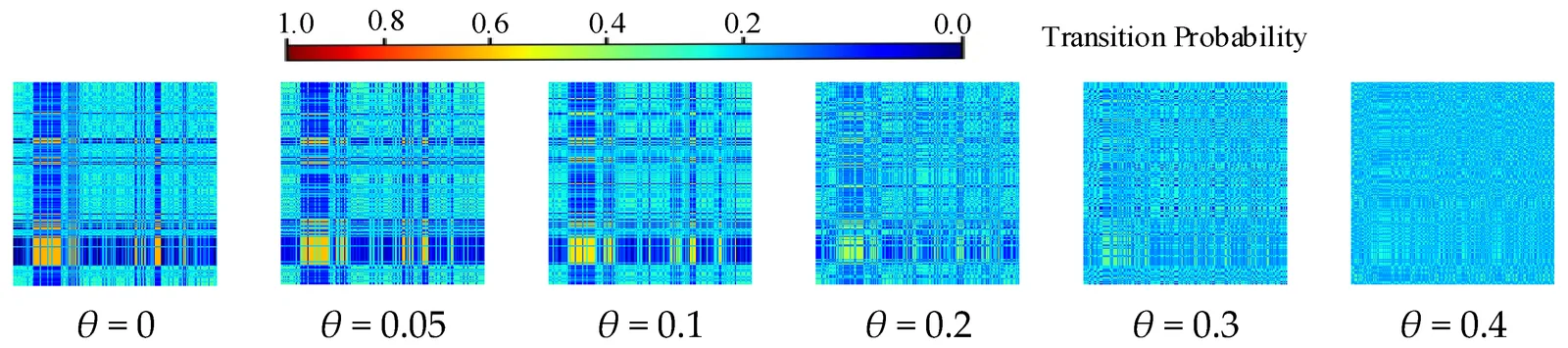
FMTF at different noise levels.

When the Gaussian noise intensity a ranges from 0.05 to 0.4, the obtained FMTF is shown in Figure 23. It can be observed that as the noise intensity increases, the critical frequency bands in the original signal are gradually masked by noise.

**Figure 24:**
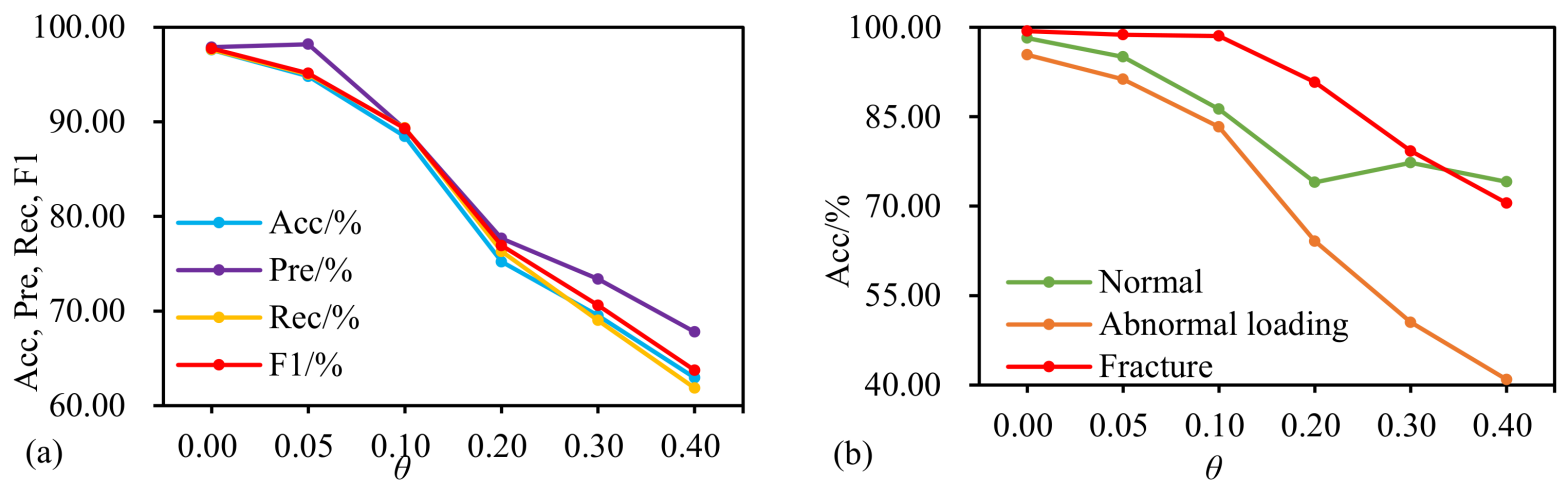
Influence of different noise levels on model performance: (**a**) Overall model recognition performance; (**b**) Acc of different classifications.

Subsequently, Gaussian noise with varying intensities was injected solely into the test set of the shear hinge state dataset without retraining the model, generating test subsets under distinct noise levels. The model trained under the zero-noise condition was adopted to test these noisy test subsets. The average results of five repeated experiments were taken as the final evaluation metrics, and the corresponding test outcomes are presented in Figure 24.

As shown in Figure 24, the overall recognition accuracy of the model declines continuously with the increase of Gaussian noise intensity, since the key frequency bands are obscured by noise. When a rises to 0.2, all overall evaluation metrics degrade significantly. Nevertheless, the recognition accuracy for shear hinge fracture remains above 75%, considerably higher than that for the normal and abnormal-response working conditions. Even when a reaches 0.4, the fracture identification accuracy still remains at 70%.

2. Influence of Impulse Noise on Model Performance

**Figure 25:**
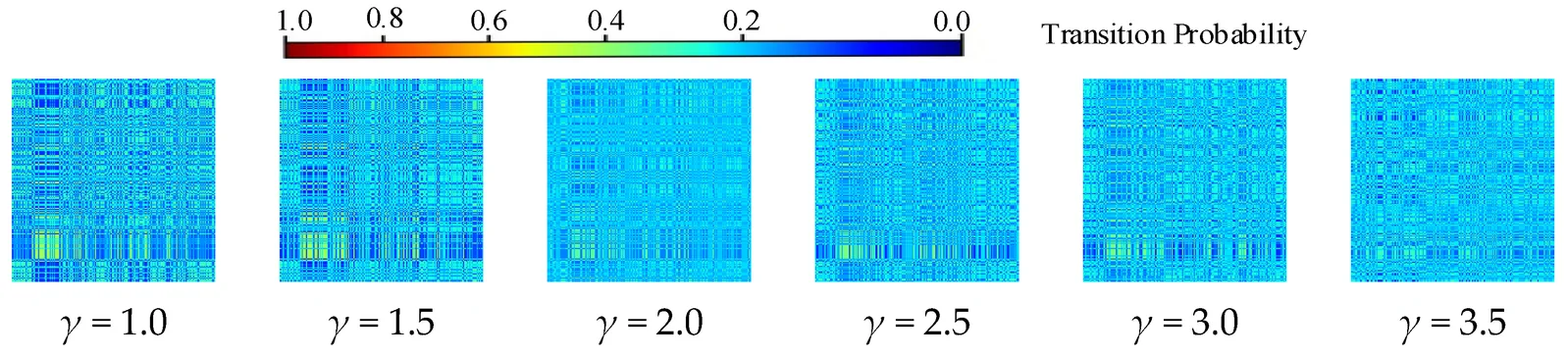
FMTF at different γ.

The occurrence probability of impulse noise *ρ* takes the values of [0.01, 0.03, 0.05, 0.08, 0.1], and the amplitude multiplier of impulse spikes *γ* is set to [1.0, 1.5, 2.0, 2.5, 3.0, 3.5]. The obtained FMTF at *ρ* = 0.05 is presented in Figure 25. It can be observed that as the noise intensity increases, the critical frequency bands of the original signal are gradually masked by noise.

**Figure 26:**
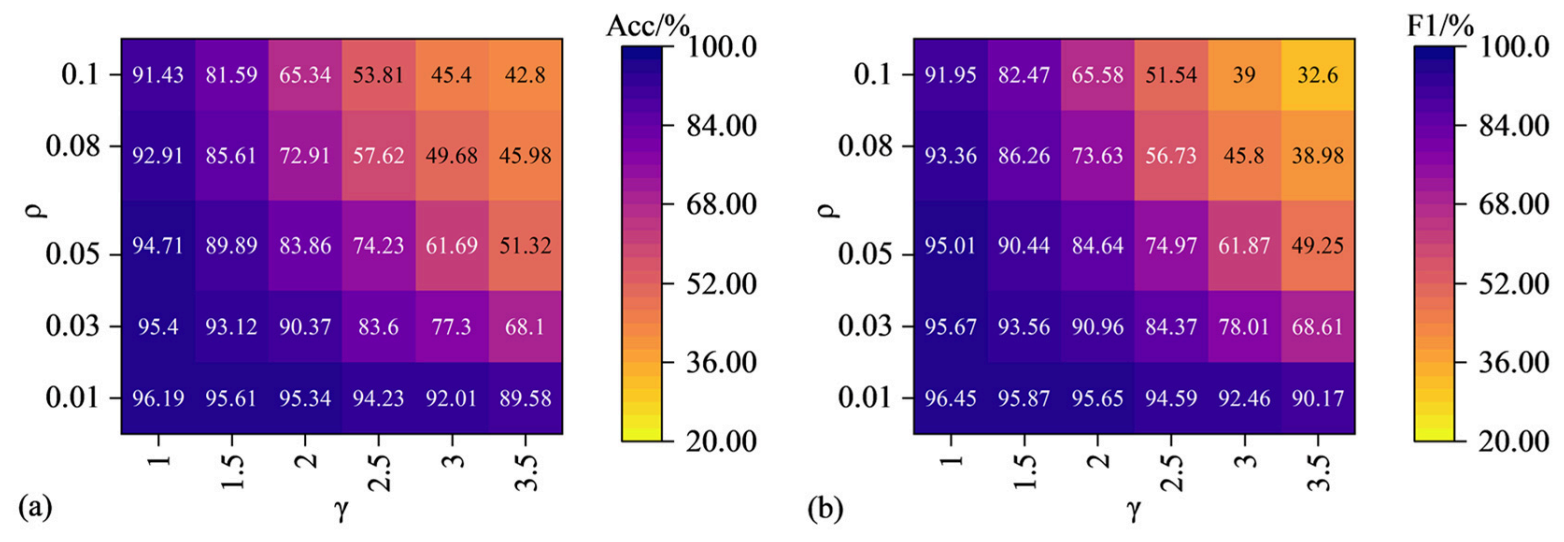
Influence of different impact noise levels on model performance: (**a**) Acc; (**b**) F1.

Subsequently, on the shear-hinge condition test set, impulse noise is added only to the original signals without retraining the model to obtain test sets under different noise levels. The model trained under zero-noise conditions is adopted to evaluate test sets with different occurrence probabilities and amplitude multipliers. The mean value of five repeated experiments is regarded as the final evaluation result, and the test results are shown in Figure 26.

As can be seen from Figure 26, when *ρ* = 0.01, the model still achieves an accuracy of 89.58% and maintains high recognition accuracy even if *γ* increases to 3.5. When *ρ* rises to 0.03, both accuracy and F1-score remain above 90% provided that the amplitude multiplier *γ* does not exceed 2.0. Model performance gradually degrades with the further increase of *ρ*. Even under the moderate noise probability *ρ* = 0.05, the F1-score can still reach 84.64% for *γ* < 2, which corresponds to an acceptable recognition level. Obvious performance deterioration occurs only when *ρ* = 0.05 together with *γ* > 2. The above results demonstrate that the proposed model possesses strong resistance to impulse noise with low-to-moderate occurrence probability. Significant performance decline takes place only when impulse noise appears frequently at high probability, which indicates the application potential of the model under complex noisy conditions in practical track-site scenarios.

#### 4.3.8. Influence of Different Metro Lines on Feature Extraction of Shear Hinges

**Figure 27:**
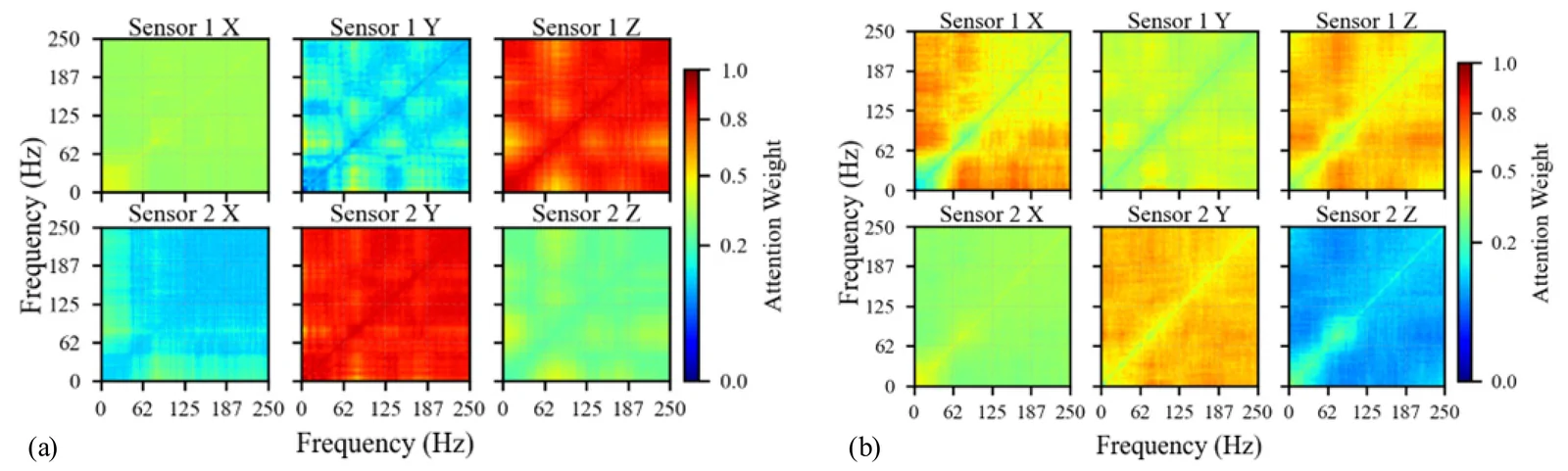
2DCNN attention map: (**a**) Line 5; (**b**) Mixed shear hinge samples from all lines.

Fault features of shear hinges vary greatly across different metro lines. Using identical-parameter FMTF-PCNN-RWKV, experiments with single-line and multi-line samples are performed to investigate line effects on feature extraction. Averaging attention maps of 50 samples yields the FMTF attention distribution for each component, whose visualization on six 2DCNN branches is given in Figure 27.

The study reveals that under single-track operating conditions, the fault-sensitive vibration axis varies with the specific line environment and structural transmission characteristics, and the model focuses on the optimal single-sensor channel. After multi-track mixed training covering 11 routes, all six X/Y/Z sensor channels exhibit high-weight diagonal frequency bands, which eliminates feature bias induced by single-track data and realizes multi-dimensional feature fusion of all sensors. Although the axial weights differ across various lines, PCNN-RWKV can consistently extract valid features above 60 Hz, demonstrating that the proposed model is capable of capturing natural frequency shifts caused by hinge faults.

#### 4.3.9. Influence of Signal Loss on Model Recognition Performance

To verify the impact of missing signals on model performance, several branches of the well-trained FMTF-PCNN-RWKV model are disabled. Corresponding tests are directly carried out on the test set, and the average results of five repeated experiments are taken as the final evaluation metrics, as listed in Table 6.

It can be concluded from Table 6 that the single-axis and dual-axis signal loss experiments demonstrate that all three vibration components of the two sensors contain effective feature information for identification. Among them, the Y-axis signals of both sensors are the most critical for shear hinge state recognition, followed by the X-axis and Z-axis signals. Model performance presents a stepwise rising trend as the number of input signal components increases. Experimental groups no. 19 and no. 20 reveal that the model fails to accurately judge shear hinge conditions when only the signals from Sensor 1 or Sensor 2 are used independently. The results indicate that integrating all features from the three components of both sensors is required to achieve superior recognition accuracy. When one axis of signal data is missing, the model accuracy only decreases by approximately 2%. Even under the loss of two axes of signals, the recognition accuracy remains around 80%, which further verifies the strong robustness of the proposed model.

#### 4.3.10. Influence of Signal Disorder on Model Recognition Performance

To investigate how signal disorder affects model performance, we directly shuffle the input order of test-set signals for the well-trained FMTF-PCNN-RWKV. The averaged results from five repeated experiments are reported as the final evaluation, as shown in Table 7.

As can be seen from Table 7, the input order of sensor channels significantly affects model performance. After shuffling the channel arrangement, most disorder-perturbed experimental groups suffer an obvious accuracy drop, with the accuracy decreasing to approximately 40%. Although several disordered combinations show relatively mild degradation, their accuracy is still markedly lower than that under the original ordered input. This indicates that RWKV is sensitive to the position of input sequences. It implicitly leverages sequence position encoding to build dependencies among features from different channels. Once the physical meaning of each channel is misaligned with its sequence position, the inter-channel correlation learned by the model will deviate, leading to degradation of recognition performance. Compared with the sharp performance drop caused by channel disorder, the performance degradation induced by the loss of a small number of sensor channels is relatively slight in the signal-missing experiments. It indirectly demonstrates that the model is more sensitive to channel arrangement order than to the loss of a single channel.

#### 4.3.11. Comparison with Other Models

To further verify the effectiveness of the proposed model, its performance is compared with classic neural networks, including LeNet, AlexNet, ResNet18, MobileNet, EfficientNet, DenseNet, and PCNN-Transformer. For Transformer, the number of encoder layers is set to 2, the number of attention heads is 4, the embedding dimension is 400, and position encoding is disabled. To ensure fair comparison among different models, all comparative models adopt the identical multi-branch input scheme. The six-channel FMTF images from sensors are neither channel-stacked nor spatially spliced. Six network instances are instantiated: each network processes the image from one single sensor channel, extracts features, concatenates feature vectors of each branch, and finally outputs classification results via a shared classification head. This configuration guarantees equivalent input information for all models and avoids unfair factors caused by modifying network input channels or spatial image splicing. On the shear-hinge condition dataset, the same training hyperparameters as PCNN-RWKV are adopted. The mean value of five test-set runs is taken as the final evaluation result, as shown in Table 8.

As shown in Table 8, the proposed PCNN-RWKV achieves a maximum FPS of 156.56, corresponding to an inference latency of only 6.39 ms per frame. ResNet18 and PCNN-Transformer also attain high accuracy exceeding 95%. In terms of model efficiency, the ELAN-F module of PCNN-RWKV leads to a sharp rise in GFLOPs and parameter count, yet it brings substantial performance improvements. Compared with mainstream lightweight models MobileNet and EfficientNet in Table 8, the proposed model incurs only a slight increase in computational cost while attaining over 11% higher accuracy. When benchmarked against ResNet18, Transformer, and other counterparts, it exhibits prominent advantages in computational overhead and parameter volume. By contrast, PCNN-RWKV reduces the computational complexity to 0.52 GFLOPs and shrinks the model size to 0.97 M parameters, thereby accelerating the inference speed. With a single-frame inference latency of merely 6.39 ms, PCNN-RWKV significantly outperforms other models and strikes an optimal balance between high prediction accuracy and low resource consumption.

From the above analysis, the FMTF-based PCNN-RWKV shear hinge state recognition model not only attains optimal recognition accuracy but also strikes a favorable balance between model compactness and inference speed, verifying its effectiveness for practical engineering deployment.

## 5. Conclusions

In view of the complex service conditions of shear hinges in FST, the difficulty in directly acquiring their physical characteristics, and the inability of traditional signal processing methods to characterize the state features of such non-rotating components, resulting in problems such as difficulty in effective feature extraction, low accuracy, and poor noise resistance in state recognition models, this paper proposes an FMTF-based PCNN-RWKV model for shear hinge state recognition, with the following conclusions:

(1) This study constructs an FMTF-based PCNN-RWKV method for shear hinge condition monitoring. By adopting the FMTF, the proposed method enhances frequency-domain correlation characteristics and enables automatic multi-scale feature extraction from multi-component FMTF images. The RWKV module adaptively establishes latent dependencies among different multi-component FMTF features. The FMTF effectively characterizes the wideband (over 60 Hz) frequency-domain variations of shear hinges under diverse health states. The developed PCNN-RWKV model achieves an overall recognition accuracy of 97.62%. With a compact structure, it efficiently extracts multi-scale features from dual-sensor and multi-component FMTF data. It maintains stable recognition performance under strong noise interference and signal loss conditions, providing an effective early warning solution for shear hinge fracture monitoring of FST.
(2) The FMTF-based PCNN-RWKV model shows significant differences in key attention regions for information from different sensors and axes. When the shear hinge state changes, the vibration signal spectra of the two floating slabs vary notably near frequencies above 60 Hz. The key attention regions of the model are broad and scattered rather than concentrated around a specific frequency, which is the most prominent difference from state recognition of rotating machinery, verifying the interpretability and effectiveness of FMTF.
(3) Combined with the working principle of shear hinges and the feature distribution of different shear hinge states extracted by the FMTF-based PCNN-RWKV model, an obvious state transition process is found between the normal working state and the abnormally stressed state of shear hinges, with a blurred boundary. This indicates that the normal and abnormally stressed states of shear hinges are mainly related to the magnitude of the force applied. In contrast, the shear hinge fracture state exhibits a clear data distribution boundary from the previous two states, demonstrating that the dynamic characteristics of the FST at the fracture position change significantly after shear hinge fracture, leading to distinct differences from the other two states.
(4) To prevent shear hinge fracture, upon detection of abnormal-response conditions, the vibration isolator support states of the floating slabs attached to both ends of the hinge should be adjusted in a timely manner, or the isolators should be replaced. Such corrective actions relieve the abnormal response on the shear hinge and guarantee the safe and stable operation of the floating slab track.

Although the shear hinge condition monitoring method proposed in this paper has achieved preliminary progress in recognition performance and engineering adaptability, there still exist directions for further expansion and in-depth research. Combined with the development trend of intelligent operation and maintenance for rail transit, future research can be carried out in the following aspects: (1) The current model results are obtained under the working-condition settings of the dataset in this study. Sensor installation tolerances, temperature-induced stiffness drift, wide-range speed variations, and cross-line environmental differences have not yet been fully quantitatively validated. Future work will focus on constructing multi-working-condition test datasets or multi-line collected datasets, conducting dedicated robustness evaluations for the above-mentioned influencing factors, and further improving the engineering applicability of the proposed monitoring method. (2) Aiming at the domain shift problem of shear hinge data in practical engineering, it is planned to combine domain adaptive learning to improve the generalization ability of the shear hinge monitoring model. (3) Future work will extend from the current condition recognition to full-life-cycle health management, and integrate degradation trend analysis and remaining useful life prediction algorithms to construct a framework for health condition assessment and life early warning of shear hinges.

## Figures and Tables

**Table 1:** Force-measurement results of isolators on both sides of the shear hinge.

Id	SVI 1 (KN)	SVI 2 (KN)	Difference	Classification
1	25.3	16.1	9.2	Abnormal-response
2	18.8	9.5	9.3	Abnormal-response
3	9.9	18.8	8.9	Abnormal-response
4	17.9	10.1	7.8	Abnormal-response
5	16.6	27.4	10.8	Fracture
6	30.2	27.0	3.2	Normal
7	21.1	26.9	5.8	Abnormal-response
8	17.7	5.5	12.2	Fracture
9	13.3	17.2	4.9	Abnormal-response
10	27.2	23.8	3.4	Normal
11	18.6	14.7	3.9	Normal

**Table 2:** Shear hinge state dataset.

Classification	Number of Shear Hinges	Training Set	Validation Set	Test Set	Sum
Normal	58	3850	825	825	5500
Abnormal-response	45	2730	585	585	3900
Fracture	8	2800	200	200	3200
Sum	111	8820	1890	1890	12,600

**Table 3:** PCNN-RWKV ablation test.

Method			Evaluation Metric					
PCNN		RWKV						
Spatial Attention	ELAN-F		Acc/%	Pre/%	Rec/%	F1/%	GFLOPs	Params/M
√	×	×	80.90	81.49	81.29	81.40	0.09	0.16
Two stacked 2DCBS		×	86.56	87.04	86.56	86.62	1.01	1.28
×	√	×	91.22	92.09	91.42	91.68	0.50	0.84
√	√	×	93.81	94.47	94.00	94.21	0.51	0.93
√	×	√	96.24	96.25	96.21	96.23	0.50	0.84
√	√	√	97.62	97.89	97.65	97.76	0.52	0.97

**Table 4:** Quantitative evaluation of T-SNE distribution of each module characteristics.

Features	Silhouette	Davies–Bouldin	k-NN Acc/%
FMTF	0.025	8.259	90.33
PCNN	0.035	6.488	96.00
RWKV	0.874	0.181	96.55
Full connection layer	0.773	0.349	96.67

**Table 5:** Comparison of recognition performance of different coding methods.

Method	Acc/%	Pre/%	Rec/%	F1/%	Time Complexity
FFT	93.25	94.85	93.26	94.03	O(*N* × log*N*) = 14,408
TMTF	76.93	77.33	77.30	76.33	O(*S*^2^) = 62,500
TGAF	82.28	82.24	82.28	81.96	O(*S*^2^) = 62,500
CWT	82.01	86.79	82.56	84.62	O(*S* × *N*) =1,000,000
STFT	97.04	97.41	97.08	97.23	O(105 × *L* × log*L*) = 215,040
FGAF	95.03	94.81	94.82	94.81	O(*N* × log*N* + *S*^2^) = 110,500
FMTF	97.62	97.89	97.65	97.76	O(*N* × log*N* + *S*^2^) = 110,500

**Table 6:** FMTF-PCNN-RWKV ablation test.

ID	Input signal						Evaluation Metric			
	Sensor 1			Sensor 2			Acc/%	Pre/%	Rec/%	F1/%
	X	Y	Z	X	Y	Z				
1	√	×	×	×	×	×	56.51	58.84	51.58	48.79
2	×	√	×	×	×	×	53.92	69.59	51.01	42.55
3	×	×	√	×	×	×	53.07	44.25	46.13	39.86
4	×	×	×	√	×	×	55.98	43.65	50.27	43.75
5	×	×	×	×	√	×	59.21	61.18	55.43	54.95
6	×	×	×	×	×	√	56.93	60.12	52.59	53.47
7	√	√	×	×	×	×	74.50	77.52	73.45	74.11
8	×	√	√	×	×	×	77.51	79.72	80.34	79.45
9	√	×	√	×	×	×	66.72	69.32	64.09	62.93
10	×	×	×	√	√	×	72.28	74.71	72.20	70.43
11	×	×	×	×	√	√	70.63	76.76	66.80	68.68
12	×	×	×	√	×	√	68.15	74.01	65.13	65.64
13	√	×	×	√	×	×	70.11	73.19	68.83	68.64
14	×	√	×	×	√	×	78.89	81.59	77.69	78.53
15	×	×	√	×	×	√	71.27	77.68	68.85	68.84
16	√	√	×	√	√	×	79.05	79.09	80.30	79.35
17	×	√	√	×	√	√	90.26	91.66	90.29	90.77
18	√	×	√	√	×	√	83.33	86.92	82.45	83.63
19	√	√	√	×	×	×	73.39	80.35	71.98	70.01
20	×	×	×	√	√	√	77.51	82.03	75.96	76.30
21	×	√	√	√	√	√	95.77	96.02	96.18	96.09
22	√	×	√	√	√	√	95.82	96.33	95.85	96.07
23	√	√	×	√	√	√	95.29	96.54	94.94	95.54
24	√	√	√	×	√	√	96.30	96.41	96.67	96.53
25	√	√	√	√	×	√	95.50	96.28	95.41	95.78
26	√	√	√	√	√	×	96.14	96.26	96.60	96.40
27	√	√	√	√	√	√	97.62	97.89	97.65	97.76

**Table 7:** Results regarding the influence of signal disorder on recognition performance of FMTF-PCNN-RWKV.

Id	Input Signal Sequence	Acc/%	Pre/%	Rec/%	F1/%
0	{0: ‘Sensor-1_X’, 1: ‘Sensor-1_Y’, 2: ‘Sensor-1_Z’, 3: ‘Sensor-2_X’, 4: ‘Sensor-2_Y’, 5: ‘Sensor-2_Z’}	95.61	95.80	96.37	95.95
1	{0: ‘Sensor-2_X’, 1: ‘Sensor-1_X’, 2: ‘Sensor-2_Z’, 3: ‘Sensor-1_Y’, 4: ‘Sensor-2_Y’, 5: ‘Sensor-1_Z’}	42.91	49.28	41.31	38.92
2	{0: ‘Sensor-1_X’, 1: ‘Sensor-1_Y’, 2: ‘Sensor-2_Z’, 3: ‘Sensor-1_Z’, 4: ‘Sensor-2_Y’, 5: ‘Sensor-2_X’}	57.20	67.58	57.43	57.26
3	{0: ‘Sensor-2_Y’, 1: ‘Sensor-2_Z’, 2: ‘Sensor-2_X’, 3: ‘Sensor-1_X’, 4: ‘Sensor-1_Z’, 5: ‘Sensor-1_Y’}	40.26	52.22	40.70	38.13
4	{0: ‘Sensor-2_X’, 1: ‘Sensor-2_Y’, 2: ‘Sensor-1_Z’, 3: ‘Sensor-1_X’, 4: ‘Sensor-1_Y’, 5: ‘Sensor-2_Z’}	74.60	80.79	74.72	75.05
5	{0: ‘Sensor-2_X’, 1: ‘Sensor-1_X’, 2: ‘Sensor-1_Y’, 3: ‘Sensor-2_Y’, 4: ‘Sensor-2_Z’, 5: ‘Sensor-1_Z’}	46.30	59.34	45.72	43.38
6	{0: ‘Sensor-2_X’, 1: ‘Sensor-2_Z’, 2: ‘Sensor-2_Y’, 3: ‘Sensor-1_Z’, 4: ‘Sensor-1_Y’, 5: ‘Sensor-1_X’}	37.35	49.36	37.98	33.74
7	{0: ‘Sensor-1_Z’, 1: ‘Sensor-1_X’, 2: ‘Sensor-1_Y’, 3: ‘Sensor-2_X’, 4: ‘Sensor-2_Y’, 5: ‘Sensor-2_Z’}	64.60	75.20	66.53	65.88

**Table 8:** Performance comparison of different models for shear hinge monitoring.

Method	Acc/%	GFLOPs	Params/M	FPS	Latency (ms)
LeNet	86.14	1.47	191.02	70.73	14.14
AlexNet	92.61	5.04	367.72	108.29	9.23
ResNet18	97.09	14.14	70.74	82.10	12.18
MobileNet	85.72	2.53	21.73	37.81	26.45
EfficientNet	91.56	3.22	32.44	70.73	14.14
DenseNet	87.63	21.14	48.54	10.68	93.63
PCNN-Transformer	95.23	11.67	0.70	141.40	7.07
PCNN-RWKV	97.62	0.52	0.97	156.56	6.39

## Data Availability

The experimental data used in this paper are derived from the vibration data of floating slab tracks of a certain metro line, and the authors have no authority to disclose them to the public.

## Abbreviations

The following abbreviations are used in this manuscript:

FST	Floating Slab Track
FMTF	Frequency Domain Markov Transition Field
PCNN	Parallel Convolutional Neural Network
RWKV	Receptance Weighted Key Value
ELAN	Efficient Layer Aggregation Network
VLZ	Z-weighted Vibration Level
Acc	Accuracy
Pre	Precision
Rec	Recall
F1	F1 Score
Params	Parameters
FPS	Frames Per Second
GAF	Gramian Angular Field
MTF	Markov Transition Field
CWT	Continuous Wavelet Transform
STFT	Short-Time Fourier Transform
